# Supplementary material for: The bispecific innate cell engager AFM28 eliminates CD123+ leukemic stem and progenitor cells in AML and MDS
Source: Nat Commun. 2025 Aug 21;16:7793. doi: 10.1038/s41467-025-63069-y (PMC12371032; doi:10.1038/s41467-025-63069-y)
Supplement: Supplementary file 1 — Supplementary Information [file 41467_2025_63069_MOESM1_ESM.pdf]

Supplementary Information

The bispecific innate cell engager AFM28 eliminates $CD123^+$ leukemic stem and progenitor cells in AML and MDS

Nanni Schmitt[1*], Jana-Julia Siegler[2*], Alexandra Beck[1], Thomas Müller[2], Izabela Kozlowska[2], Séverine Sarlang[2], Uwe Reusch[2], Stefan Knackmuss[2], José Medina-Echeverz[2], Joachim Koch[2], Thorsten Ross[2], Ali Darwich[3], Lea Hoppe[1], Mohammed Abba[1], Alexander Streuer[1], Stefan Klein[1], Wolf-Karsten Hofmann[1], Anna Lisa Gündner[2], Christian Merz[2], Jan Endell[2], Jens Pahl[2#] and Daniel Nowak[1#]

[1] Department of Hematology and Oncology, Medical Faculty Mannheim, Heidelberg University, Mannheim, 68167, Germany

[2] Affimed GmbH, Gottlieb-Daimler-Straße 2, 68165 Mannheim, Germany

[3] Department of Orthopedic Surgery, Medical Faculty Mannheim, Heidelberg University, Mannheim, 68167, Germany

[*]These authors contributed equally.

[#]These authors jointly supervised this work.

Prof. Dr. med. Daniel Nowak
Department of Hematology and Oncology
Medical Faculty Mannheim, Heidelberg University
Email: daniel.nowak@medma.uni-heidelberg.de
Phone: +49 (0) 621 383 4115

Dr. Jens Pahl
Affimed GmbH
Email: j.pahl@affimed.com
Phone: +49 (0) 621 56003-696

Supplementary Note 1

AFM28 binds with high affinity and selectivity to human and cynomolgus CD16A and CD123

We first evaluated the binding kinetics of AFM28 to human, cynomolgus or mouse Fcγ receptors (FcγR), including CD16 and CD32 variants, CD64, neonatal Fc receptor (FcRn) and CD123 by surface plasmon resonance, using experimental settings allowing for either monovalent or bivalent binding interaction. Similar binding to both high-affinity (158V) and low-affinity (158F) allelic variants of human CD16A in the low nanomolar (K_D= 2.60 [158V] and 2.33 nM [158F] for monovalent binding) or sub-nanomolar range (K_D= 0.30 [158V] and 0.24 nM [158F] for bivalent binding) was observed (Supplementary Table 1). Binding of AFM28 to cynomolgus CD16 antigens occurred in a comparable range (K_D= 4.09 nM for monovalent and 0.41 nM for bivalent binding). AFM28 demonstrated a strong, bivalent binding interaction to both human CD123 (K_D= 0.01 nM) and cynomolgus CD123 (K_D= 0.04 nM) (Supplementary Table 1). Overall, the bivalent binding affinity was increased compared with monovalent binding affinity for CD16A (8- to 10-fold) and CD123 (>10-fold). No monovalent binding was detected for human CD16B and there was very low binding (>2 µM) in the bivalent setting. No binding to murine homologs of CD16 or CD123 was observed, and AFM28 did not interact with other FcγR from tested species (data not shown). Binding to human and cynomolgus FcRn was observed with similar affinity (K_D= 364 pM and 238 pM, respectively), conferring IgG-like pharmacokinetic properties (data not shown).

Supplementary Methods

Construction of AFM28 and purification analysis

AFM28 was expressed and purified as described for a previously developed innate cell engager molecule.[1] Briefly, a high-affinity anti-CD16A single chain variable fragment (scFv) domain of a tetravalent bispecific IgG1scFv fusion antibody (scFv-IgAb), incorporating an effector silenced Fc-

portion was stabilized via an H44-L100 mutation,[2] which was employed to introduce an interdomain disulfide bridge. The resulting Redirected Optimized Cell Killing (ROCK) innate cell engager is specific for human CD123 and human CD16A with full cross-reactivity to cynomolgus CD123 and CD16. The molecular weight of AFM28 is 203 kDa.

Surface plasmon resonance

All interaction measurements such as binding affinity assays were performed at 37°C using a Biacore T200 instrument (GE Healthcare) equipped with a research-grade Sensor Chip CAP (Biotin CAPture Kit, GE Healthcare) pre-equilibrated in HBS-P+ (for FcRn interactions: PBS pH 6.0, 0.05% Tween 20) running buffer.

For monovalent interaction analysis, AFM28 was captured (FC2, FC4) on immobilized biotinylated human CD123-mFc.silenced/Avi-tag or biotinylated human CD16A158V-mFc.silenced/Avi-tag to a density of 20–50 response units (RU), before recombinant monomeric human CD16A158V, human CD16A158F, cynomolgus CD16 (concentration: 0 to 160 nM), human CD16BNA1 (concentration: 0 to 1000 nM) or human CD123 (concentration: 0 to 160 nM) was injected for 180 s at a flow rate of 40 µL/min and the complex was left to dissociate for 300 s at the same flow rate. Referencing was done against a flow cell (FC) without captured ligand (FC2-FC1, FC4-FC3).

For bivalent interaction analysis, biotinylated-mFc.silenced/Avi-tagged antigens (human CD16A158V, CD16A158F, CD16BNA1, CD123, cynomolgus CD16 or CD123) were captured (FC2, FC4) to a density of 120 to 200 RU, before AFM28 was injected (concentration: 0 to 60 nM for human CD16A158V, CD16A158F, CD123, cynomolgus CD16 and CD123; 0 to 1,000 nM for human CD16BNA1) for 240 s at a flow rate of 40 µL/min and the complex was left to dissociate for 300 s at the same flow rate. Referencing was done against a FC without captured ligand (FC2-FC1, FC4-FC3).

Interaction analysis of FcRn binding was performed at pH 6.0 in PBS/0.05% Tween 20; analytes and ligands were diluted in the same buffer. Biotinylated FcRn was captured to a density of approximately

10 to 20 RU (FC2 and FC4) before increasing concentrations (24.7 nM to 6000 nM) of diluted antibody were injected using multi-cycle kinetic mode (FC1−FC4), at a flow rate of 40 µL/min for 180 s, followed by dissociation for 200 s. Referencing was done against a FC without captured ligand (FC2−FC1, FC4−FC3).

Interaction analysis of AFM28 binding to human FcγRI (CD64), FcγRIIA (CD32A), FcγRIIB (CD32B), FcγRIIC (CD32C), cynomolgus FcγRI (CD64), FcγRIIA (CD32A) and FcγRIIB/C (CD32B/C), and murine FcγRI (CD64), FcγRIIB (CD32), and FcγRIV (CD16-2) was performed by capturing receptors to a density of approximately 15 to 30 RU (FC2, FC3, and FC4) before increasing concentrations (500 nM to 4000 nM) of antibody were injected, using the single-cycle kinetic mode (FC1−FC4) at a flow rate of 40 µL/min for 100 s followed by dissociation for 90 s. A zero-concentration cycle and ligand-free surface in FC1 was used for referencing of response signals (FC2-1, FC3-1 and FC4-1).

Interaction kinetics to CD16 and CD123 were determined by fitting data from multi-cycle kinetics experiments to a simple 1:1 interaction model using the local data analysis option (R_{max} and RI) available within Biacore T200 Evaluation Software (v3.1). Binding affinities to FcRn and FcγR were determined by fitting data using the steady-state affinity model of the Biacore T200 Evaluation software (v3.1). After each cycle, the chip surfaces were regenerated with 6 M guanidine-HCl, 0.25 M NaOH and reloaded with Biotin Capture reagent.

NK cell expansion

For the generation of cryopreserved, cytokine-expanded allogeneic NK cells used in the ex vivo assays with fresh AML PB and in the hIL15-NOG xenograft mouse model, NK cells were isolated from a healthy donor-derived leukopac (Cellex Cell Professionals) by means of the CliniMACS Plus instrument (Miltenyi Biotec) by depletion of $CD3^+$ cells followed by enrichment of $CD56^+$ cells using the CliniMACS® CD3 and CD56 Reagents (Miltenyi Biotec, cat. no. 200-070-113 and 200-070-128) according to manufacturer's instructions. Enriched NK cells were cultured in NK MACS expansion medium (Miltenyi Biotec, cat. no. 130-114-429) supplemented with IL-2 (500 U/mL, Sigma-Aldrich, cat. no.

11011456001) and IL-15 (1 ng/mL, Miltenyi Biotec, cat. no. 130-095-765) for 14 days in 100 M G-Rex plates. Expansion resulted in 99% NK cells ($CD56^+$ $CD3^-$ $CD19^-$ $CD14^-$ $CD45^+$ cells) consisting of 83% $CD16^+$ NK cells. NK cells were cryopreserved in CryoStor CS10 freezing medium (STEMCELL Technologies, cat. no. 07930) using the ViaFreeze instrument (Cytiva). 14-days cytokine-expanded NK cells displayed an activated phenotype as indicated by increased expression of CD56, NKp44 and NKp30, while initial induction of the early activation marker CD69 diminished over time (see Supplementary Figure 7A). Note, that the frozen NK cell expansion product used for AFM28-associated experiments throughout the manuscript (red data points) was functionally comparable to other expansions, derived from five different healthy donors using this standardized expansion protocol, in terms of phenotypes after 1 day vs 14 days of expansion, and ADCC activity induced by the CD30/CD16A engager acimtamig (AFM13) against $CD30^+$ target cells (Supplementary Figure 7A-B). Notably, one of these additional preparations (blue data points) was tested in another proof-of-concept in vivo CDX model, demonstrating increased anti-tumor activity against $CD30^+$ cells in combination with the CD30/CD16A engager AFM13 (Supplementary Figure 7C) analogous to the CD123+ CDX model for AFM28 in this manuscript.

To generate cryopreserved AFM28-armed allogeneic NK cells, after NK cell expansion and prior to cryopreservation, one portion of the NK cells ($5x10^6$/mL) was incubated with AFM28 ($5x10^5$ pM) diluted in complete RPMI medium for 30 min at 37°C. Afterwards, cells were washed twice to remove unbound AFM28 followed by cryopreservation. As a control, the second portion of NK cells was only treated with complete RPMI medium prior to cryopreservation.

Degranulation assay

CD107a upregulation on NK cells as a marker for degranulation and intracellular IFN-γ expression of NK cells was measured in singlicate after co-culture with or without EOL-1 tumor cells at 1:1 ratio (each $1×10^5$ cells) for 4 hours in the presence of anti-CD107a FITC (1/10) and GolgiPlug (1/1000, BD Biosciences, cat. no. 555029) in 96-well microtiter plates in complete RPMI medium. As indicated, co-

cultures were performed in the presence of AFM28 ($2x10^3$ pM). After 4 hours, cells were subjected to extracellular staining of NK cell surface markers, followed by intracellular staining of IFN-γ (1/100) and then measured by flow cytometry. Cultures of NK cells alone were used to define the threshold for individual percentages of CD107a-positive and IFN-γ-positive viable $CD56^+$ $CD45^+$ CD3- SSC^{low} FSC^{low} NK cells.

Cynomolgus animal welfare, care, and use statement and veterinary care/palliative and prophylactic measures

All procedures in this Study Protocol are in compliance with the German Animal Welfare Act. In the opinion of the Sponsor and Study Director, the study does not unnecessarily duplicate any previous work, and no other model can fulfill the study requirements. This study was performed in consideration of the following directives and recommendations.

- DIRECTIVE 2010/63/EU OF THE EUROPEAN PARLIAMENT AND OF THE COUNCIL of 22 September 2010 on the protection of animals used for scientific purposes Labcorp Study Number 8470025 Final Study Protocol 14
- Commission Recommendation 2007/526/EC on guidelines for the accommodation and care of animals used for experimental and other scientific purposes (Appendix A of Convention ETS 123)
- The study design was reviewed and approved by the Landesamt für Natur, Umwelt und Verbraucherschutz Nordrhein-Westfalen (LANUV).

Supplementary Figures

Supplementary Figure 1

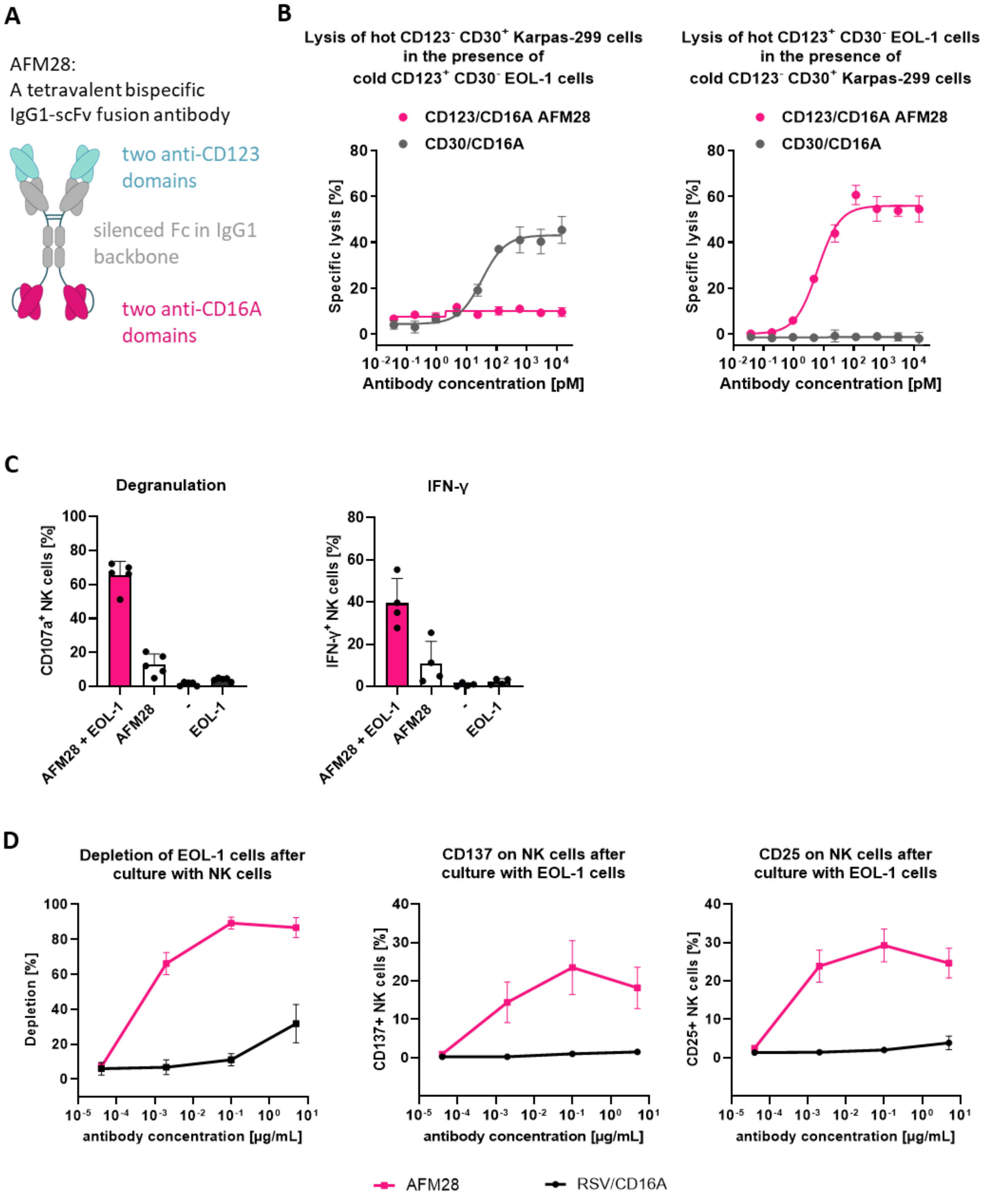

Supplementary Figure 1. Scheme of AFM28 and AFM28-mediated activation of NK cells.

A, Representative scheme of AFM28**. B,** 4 h cytotoxicity assays after co-culture of buffy coat-derived NK cells and calcein-labelled Karpas-299 cells (referred to as 'hot' targets) in the presence of unlabelled bystander EOL-1 cells (referred to as 'cold' targets), or vice versa, at an E:T ratio of 5:1:1 without and with increasing concentrations of AFM28 or an CD30/CD16A engager as indicated. One representative experiment (mean ±SD of two technical replicates) for the indicated tumor cell lysis is shown out of three performed. **C,** CD107a expression as a marker for NK cell degranulation and intracellular IFN-γ expression of NK cells was measured by flow cytometry after 4 h co-culture of NK cells without or with EOL-1 cells (E:T ratio 1:1) without or with AFM28. The gating strategy for NK cells is indicated in Supplementary Figure 8B. Data are represented as the mean ± SD of the percentage of CD107a^{+} NK cells (n=5) and IFN-γ^{+} NK cells (n=4). **D,** 24 h co-culture of NK cells with EOL-1 cells, labelled with CellTracker Green (CMFDA, 0.5 µM), at E:T 1:1 in the presence of increasing concentrations of AFM28 or RSV/CD16A control. The percentage of EOL-1 depletion was assessed by normalizing the number of CMFDA^{+} viable EOL-1 cells (negative for Annexin V and Fixable Viability Dye eFluor™ 780) to baseline (i.e., without antibody exposure or the lowest concentration of the control antibody). The gating strategy for NK cells and EOL-1 cells is indicated in Supplementary Figure 8C. Data are represented as the mean ± SD of the percentage of EOL-1 depletion (n=5), the percentage of CD137^{+} NK cells (n=5) and CD25^{+} NK cells (n=5).

Supplementary Figure 2

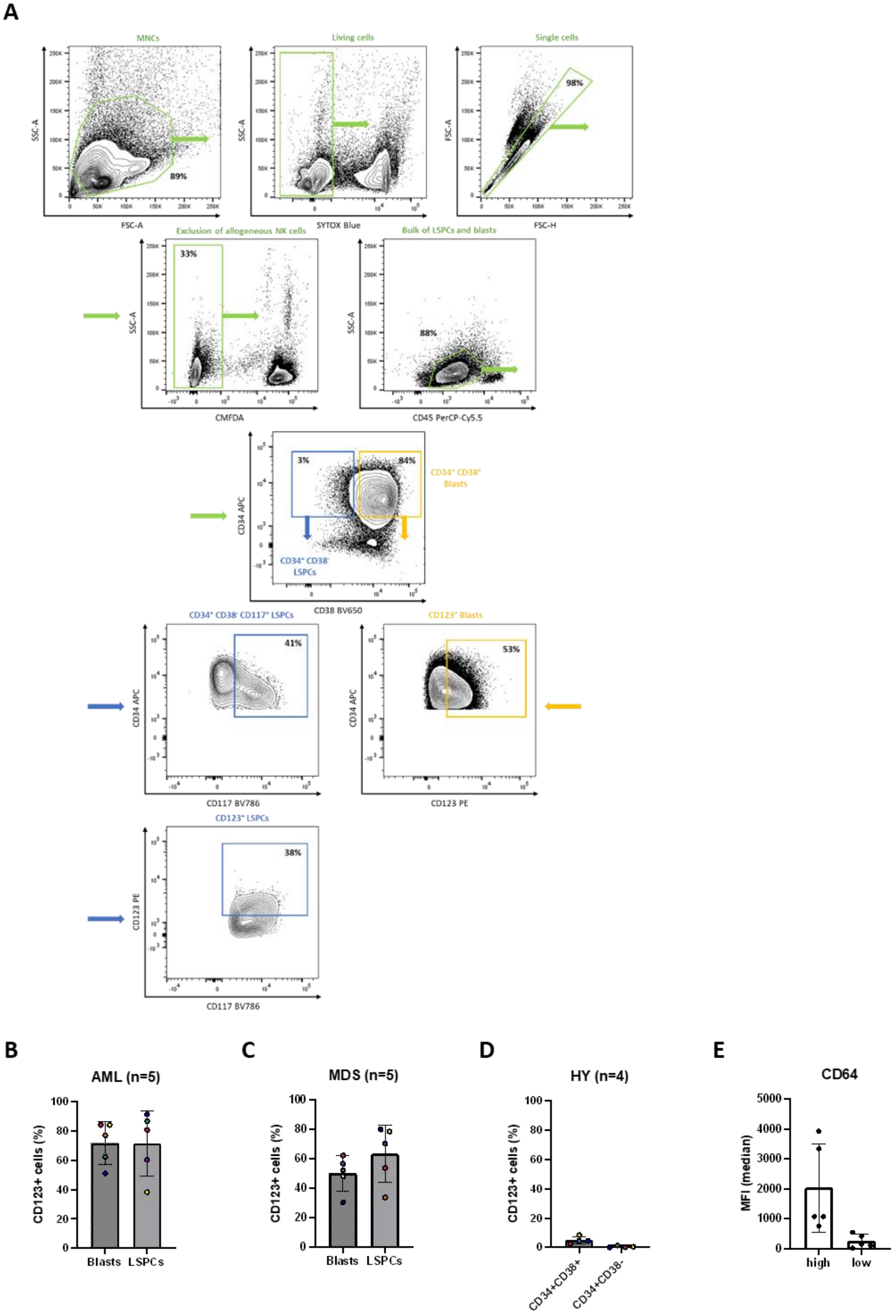

Supplementary Figure 2. Gating strategy of blasts and LSPCs, and their proportion of CD123$^+$ and CD64$^+$ cells of AML and HR-MDS patient-derived, and healthy BMMC samples.

A, Gating strategy of blasts and LSPCs. After identification of the SSC^{low}CD45^{low} population consisting of blasts and LSPCs (green gates), samples were further analyzed for CD34$^+$ CD38$^+$ or CD33$^+$ CD38$^+$ CD123$^{+/–}$ blasts (yellow gates) and CD34$^+$ CD38$^-$ or CD33$^+$ CD38$^-$ CD117$^+$ CD123$^{+/–}$ LSPCs (blue gates). The gating for CD33$^+$ blasts is not shown separately and corresponds to that of CD34$^+$ blasts. **B–D,** Percentage of CD123$^+$ cells in blast and LSPC populations of AML (**B,** n=5)**,** and MDS (**C,** n=5)**,** and CD34$^+$ CD38$^+$ and CD34$^+$ CD38$^-$ populations of healthy (**D,** n=4) BMMC samples. Data are represented as mean ± SD. Blasts were defined as viable SSC^{low} CD45^{low} CD34$^+$ CD38$^+$ and LSPCs were defined as viable CD45$^+$ CD34$^+$ CD38$^–$ CD117$^+$. **E,** Quantitative analysis of the MFI of CD64 on the AML (n=10) patient BMMC samples shown in Figure 2 determined by flow cytometry. MFI of the isotype control antibody was subtracted. MFI, median fluorescence intensity.

Supplementary Figure 3

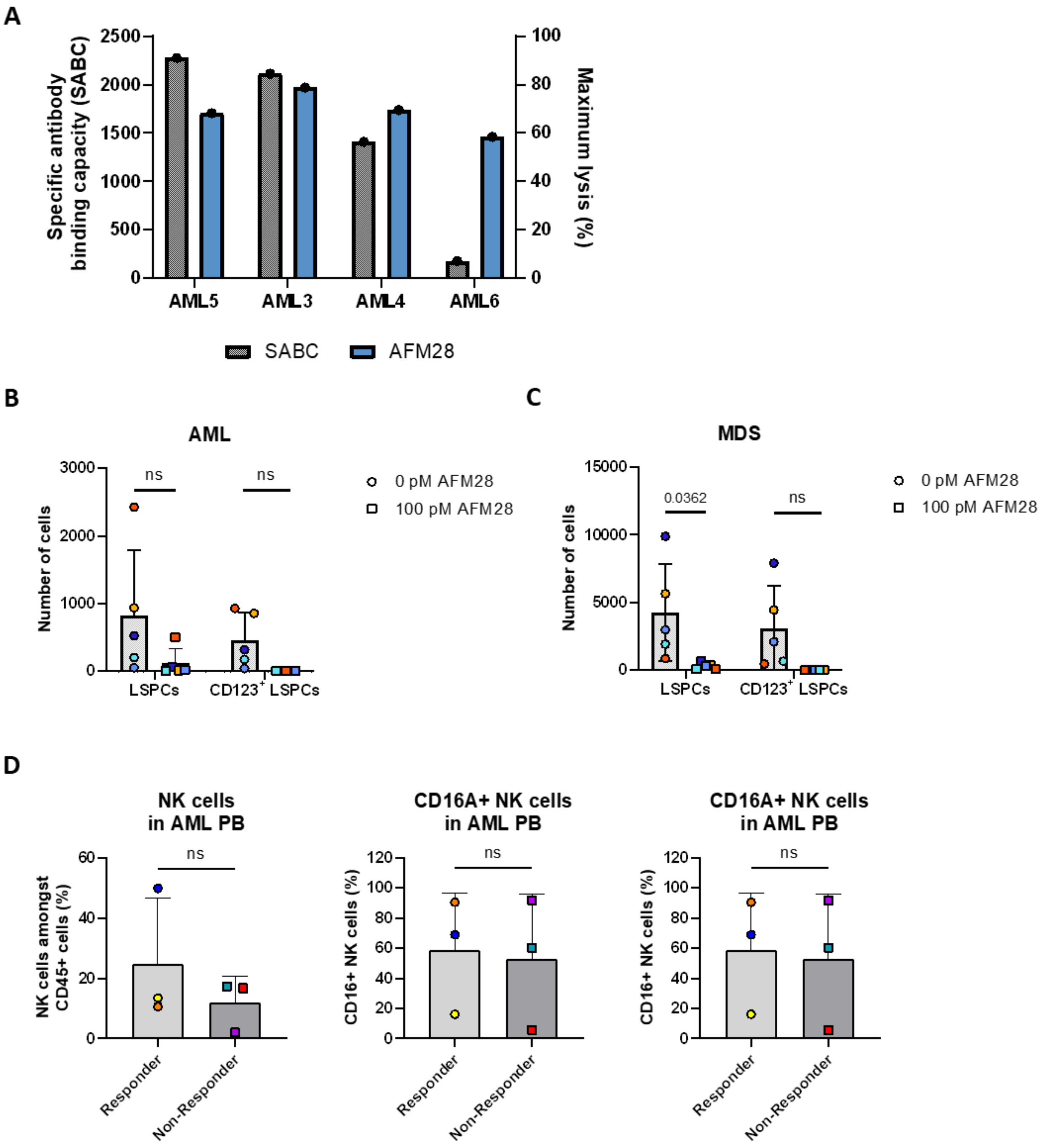

Supplementary Figure 3. AFM28 efficiently directs allogeneic NK cell cytotoxicity to $CD123^{+}$ LSPCs in AML and MDS patient samples.

A, Comparison of primary individual AML patient samples (n=4) regarding CD123 target antigen quantification on the cell surface of BMMCs (grey bars) and maximum lysis of $CD123^{+}$ blasts by AFM28 (100 pM) in the presence of allogeneic NK cells at an E:T ratio of 1:1 (blue bars), both measured by flow cytometry. Blasts were defined as viable SSC^{low} $CD45^{low}$ $CD34^{+}$ $CD38^{+}$ cells. AFM28 showed substantial lysis for samples with CD123 expression levels as low as 200 antigens per cell determined by CD123-specific antibody binding capacities (SABC). **B, C,** BMMC samples were treated with vehicle or 100 pM of AFM28 for 24 h in the presence of allogeneic NK cells at an E:T ratio of 1:1. Analysis was performed using flow cytometry. LSPCs were defined as viable $CD45^{+}$ $CD34^{+}$ $CD38^{-}$ $CD117^{+}$ cells. Reduction of cell counts of gated LSPCs from AML patients (**B,** n=5) and MDS patients (**C,** n=5). Data are represented as mean ±SD and were analyzed using one-way and two-way ANOVA. **D,** Percentage of total $CD56^{+}$ $CD3^{-}$ NK cells, and percentage and MFI of $CD16^{+}$ NK cells in peripheral blood of AML patients used for ex vivo assays. MFI of the isotype control antibody was subtracted. Individual responder patient samples were defined in Figure 4A as resulting in more than 50% leukemic blast lysis in response to AFM28. The color code of the individual patient samples matches that in Figure 4A. Data are represented as mean ±SD and were analyzed using unpaired t-test. MFI, median fluorescence intensity; ns, non-significant.

Supplementary Figure 4

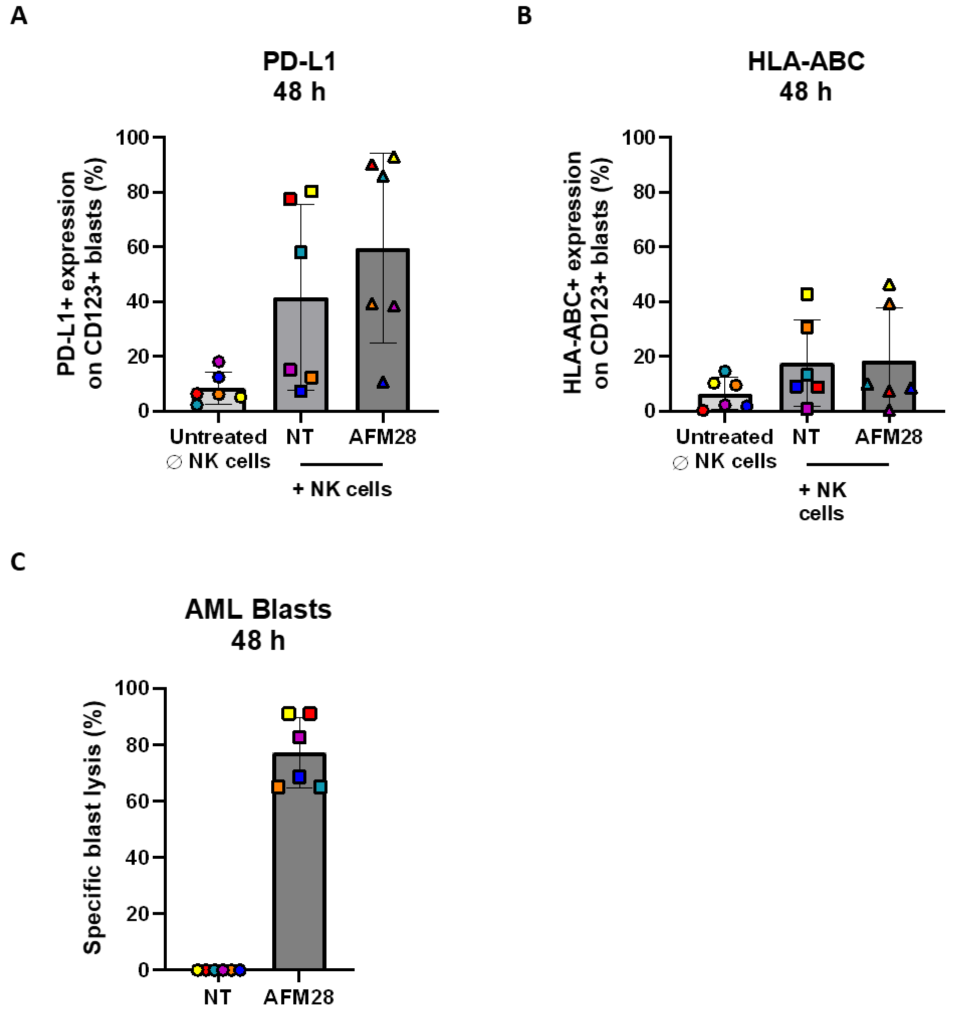

Supplementary Figure 4. Percentage of PD-L1$^+$ and HLA-ABC$^+$ CD123$^+$ blasts for different treatment conditions after 48 h ADCC assay.

A, B, Percentage of PD-L1$^+$ (**A**) and HLA-ABC$^+$ (**B**) CD123$^+$ blasts of AML BMMC samples (n=6) for the conditions untreated and without NK cells, treated with 100 pM of non-targeting (NT) control RSV/CD16A in the presence of allogeneic NK cells. Prior to the assay, non-expanded NK cells were intracellularly stained with 0.5 µM 'CellTracker Green CMFDA Dye' (Thermo Fisher) at a cell density of 10^6 cells/mL. For specific blast lysis analysis, 2.5×10^5 non-expanded allogeneic NK cells derived from healthy donors were co-cultured with 2.5×10^5 AML-BMMCs in the absence or presence of 100 pM AFM28 or a NT control in triplicates, and incubated for 48 h. Afterwards, cells were blocked with human Fc receptor blocking reagent (Miltenyi Biotec) and subjected to extracellular antibody staining

(Supplementary Table 6) to identify blasts and their expression of CD274 (PD-L1) and HLA-ABC. Dead cells were excluded using SYTOX Blue (Thermo Fisher). Analysis was performed using flow cytometry. Blasts were defined as viable/CD45^{low}/CD34^{+} or CD33^{+}/CD38^{+}/CD123^{+} cells. **C,** Lysis of CD123^{+} blasts from the AML BMMC samples phenotyped for their percentage of PD-L1^{+} (**A**) and HLA-ABC^{+} (**B**) cells. Cell counts of NT condition were set to baseline. Data are represented as mean ± SD. The color code of individual patients is identical in each graph.

Supplementary Figure 5

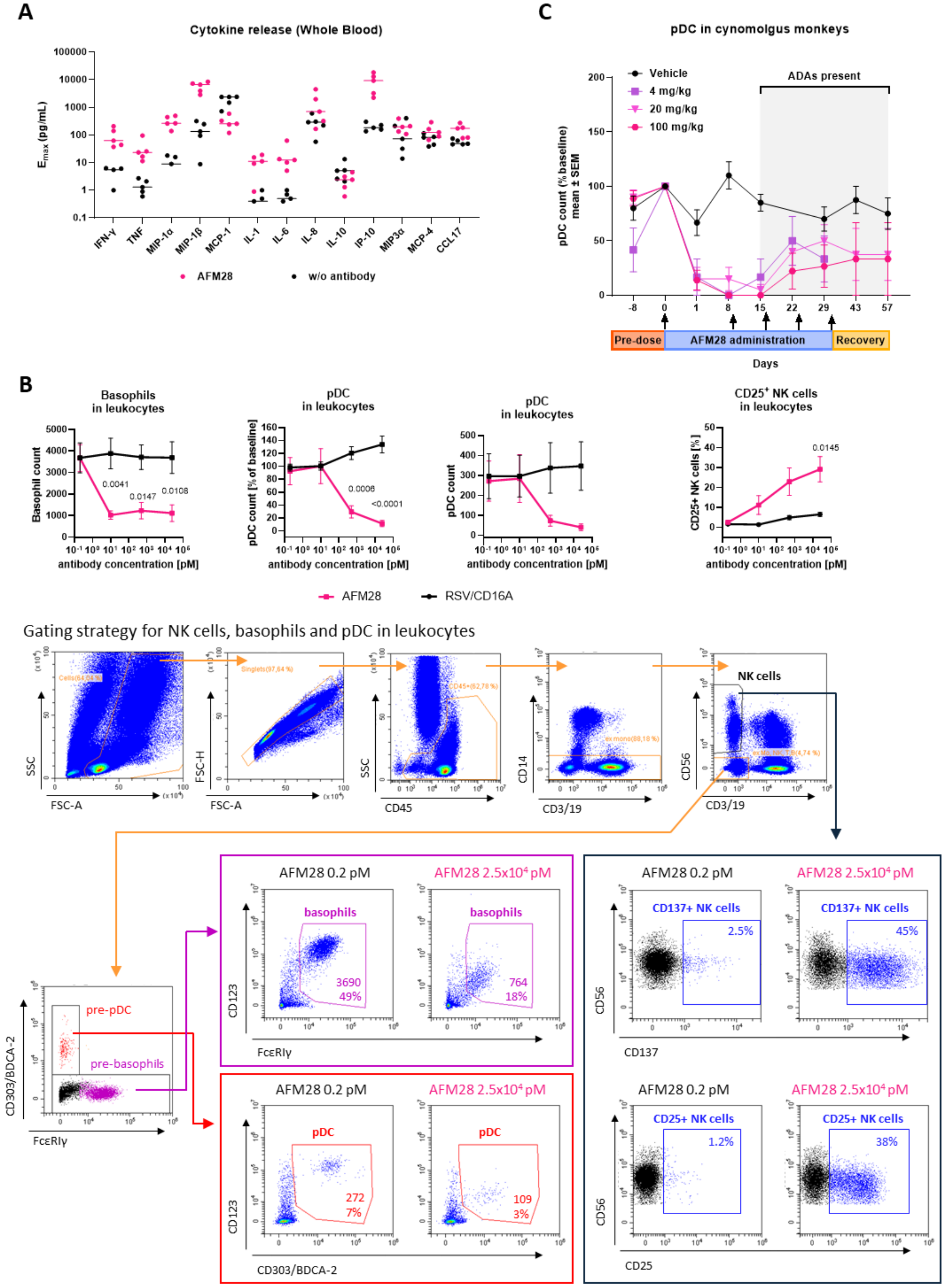

Supplementary Figure 5. AFM28 induced low cytokine release in whole blood and depletion of CD123⁺ target cells in vitro and in vivo.

A, Circulating healthy donor whole blood containing endogenous CD123^{+} target cells (basophils, pDCs). Comparison of cytokine release in whole blood treated with AFM28 (1 µg/mL) or without antibody (w/o) after 24 h incubation. E_{max} denotes maximal induction of the respective cytokine. Cytokines were measured using MSD human 36-plex array. Individual data from five donors are shown. **B,** Primary human leukocytes were incubated with increasing concentrations of AFM28 or RSV/CD16A control engager for 24 h. Data depict the mean ±SD of six donors of the dose-dependent depletion of basophils and pDC (depicting percentage of remaining cells from baseline and/or corresponding raw cell counts) and the dose-dependent changes in CD25 expression on NK cells. Depletion was assessed by flow cytometry by normalizing the number of viable (negative for Fixable Viability Dye eFluor™ 780) CD3/19/14/56/CD303^{-} FcεR1α^{+} basophils and CD3/19/14/56/FcεR1α^{-} CD303^{+} pDC to baseline (i.e., without antibody exposure or the lowest concentration of the control antibody), referred to as percentage of remaining cells from baseline, or by depicting corresponding raw cell counts. Data were analyzed using two-way ANOVA and Šídák's multiple comparisons test. Flow cytometry dot plots illustrate the percentage and raw counts of basophils and pDC (including CD123 expression) and the percentage of CD56^{+} NK cells positive for CD137 or CD25 at the highest and lowest AFM28 concentrations. **C,** pDC depletion in cynomolgus monkeys gated as CD3/CD14/CD20/CD159a/FcεR1a^{-} HLA−DR^{+} CD303^{+} cells as described in Figure 7. Data are represented as mean ±SD (10 animals per vehicle, 20 mg/kg and 100 mg/kg groups; 6 animals per 4 mg/kg group).

Supplementary Figure 6

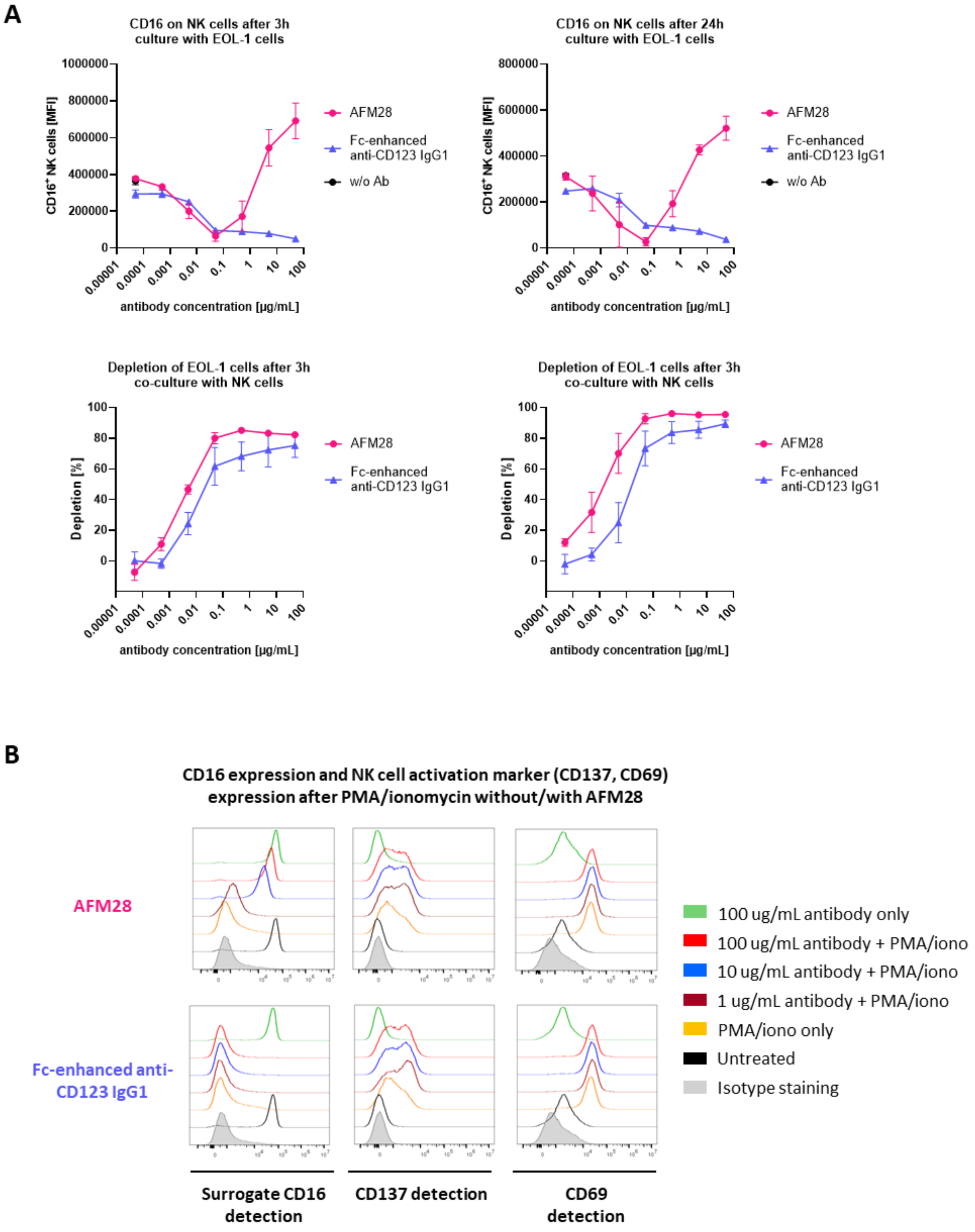

Supplementary Figure 6. AFM28 prevents PMA/Ionomcyin-induced CD16A shedding.

A, Expression of CD16A on NK cells and corresponding depletion of EOL-1 cells (see Supplementary Figure 1) after 3 h and 24 h co-culture with EOL-1 target cells (E:T 1:1) in the presence of increasing concentrations of AFM28 or Fc-enhanced anti-CD123 IgG1 or without antibodies. The gating strategy for NK cells and EOL-1 cells is indicated in Supplementary Figure 8C. Data are represented as mean ±SD of two independent experiments. **B,** NK cells were left untreated or loaded with increasing concentrations of AFM28 (1/10/100 µg/mL equivalent to $5x10^3$/$5x10^4$/$5x10^5$) or Fc-enhanced anti-CD123 IgG1 (1/10/100 µg/mL equivalent to $7x10^3$/$7x10^4$/$7x10^5$ pM) for 45 min on ice. After extensive washing, when indicated, NK cells were exposed to PMA (50 ng/mL) and ionomcyin (0.5 µM) for 4 h at 37 °C. Afterwards, surrogate CD16A expression was detected by saturated loading with AFM28 or Fc-enhanced anti-CD123 IgG1 (both 100 µg/mL) followed by goat anti-human Fc-specific secondary antibody alongside detection of the NK cell activation markers CD137 and CD69. The gating strategy for NK cells is indicated in Supplementary Figure 8D. Data are representative of three independent experiments.

Supplementary Figure 7

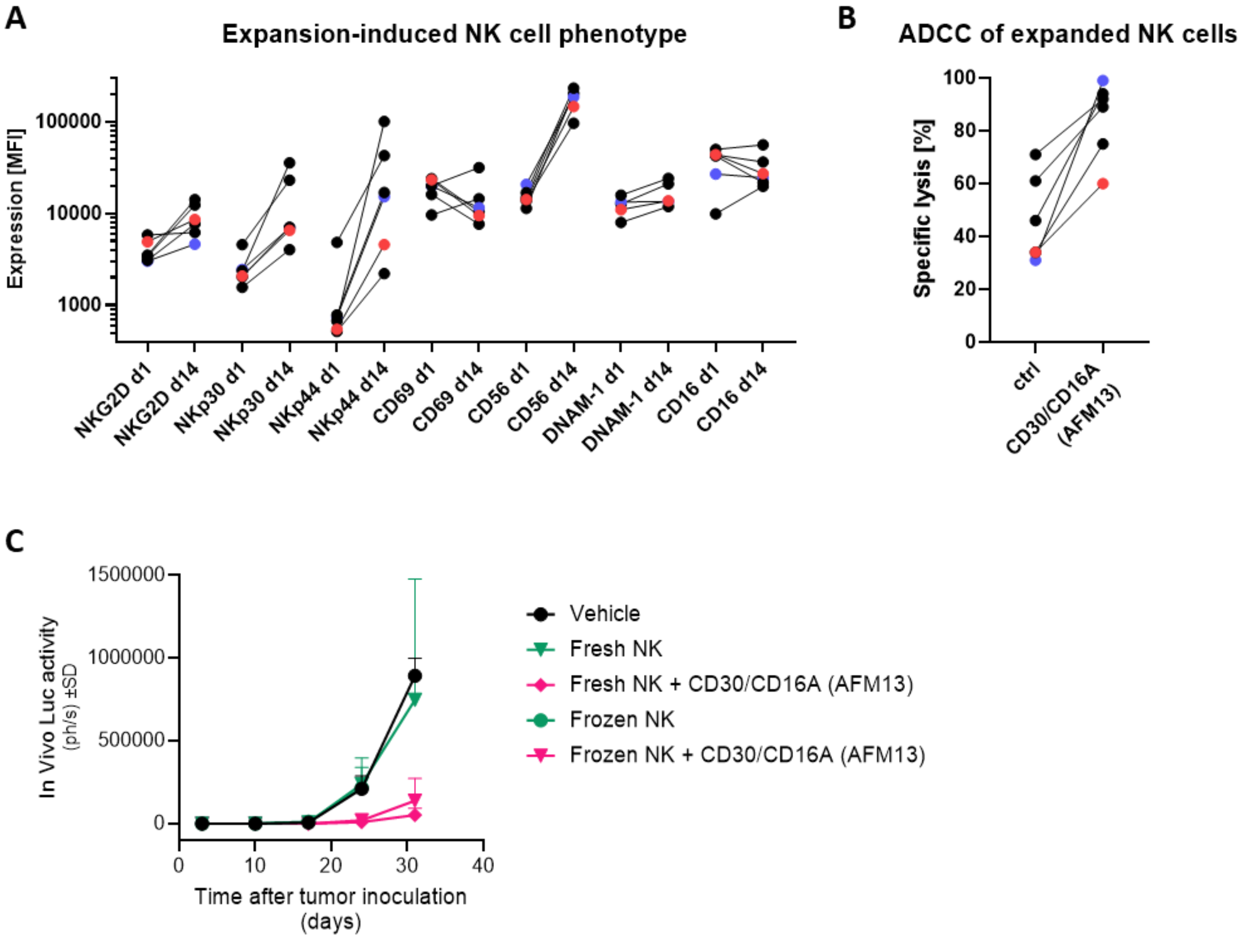

Supplementary Figure 7. Comparative overview of NK cell expansions.

A-B, Six preparations of cytokine-expanded NK cells, derived from six healthy donor leukopacs, following a standardized protocol (see Supplementary Methods), were tested for their **(A)** expression of NK cell receptors immediately after 1 day and 14 days of expansion and **(B)** ADCC activity induced by the CD30/CD16A engager acimtamig (AFM13) against $CD30^+$ Karpas-299 target cells. Data show the individual data points for the six NK cell preparations. For comparability, the NK cell expansion product used for AFM28-associated experiments throughout the manuscript is indicated by red data points. Blue data points indicate an NK cell preparation that was additionally tested in another proof-of-concept in vivo CDX model, demonstrating increased anti-tumor activity against $CD30^+$ cells in combination with the CD30/CD16A engager AFM13 (see below), analogous to the $CD123^+$ CDX model

for AFM28 (Figure 6D-E). **C**, On day 0, 10-week-old female hIL-15 NOG mice (n=40 mice, 8 mice/group) received 1.2 Gy irradiation, followed by injection IV of 1×10^5 Karpas-299_Luc cells four hours later. Immediately after tumor cell inoculation on day 0, the mice were treated IV with expanded NK cells ($1\text{x}10^7$), either fresh or frozen. Treatment with fresh NK cells was performed once at study start (d0), while treatments with frozen NK cells were performed on d0 and continued twice weekly for 3 weeks. Mice were injected IV with vehicle or AFM13 (10 mg/kg) on d0 and d1, and then twice weekly. When administered at the same day, NK cell and vehicle/AFM13 injections were performed directly after one another into separate tail veins. Mice received intraperitoneal injection of IL-2 (10,000 IU, Proleukin) on d0 and continued every second day. BLI measurements were performed every week starting day 3. Tumor growth represented by in vivo luciferase expression upon treatment is shown as photons per second in a linear scale. Data are represented as mean ±SD.

Supplementary Figure 8

A Gating strategy for basophils in whole blood (see Figure 7C)

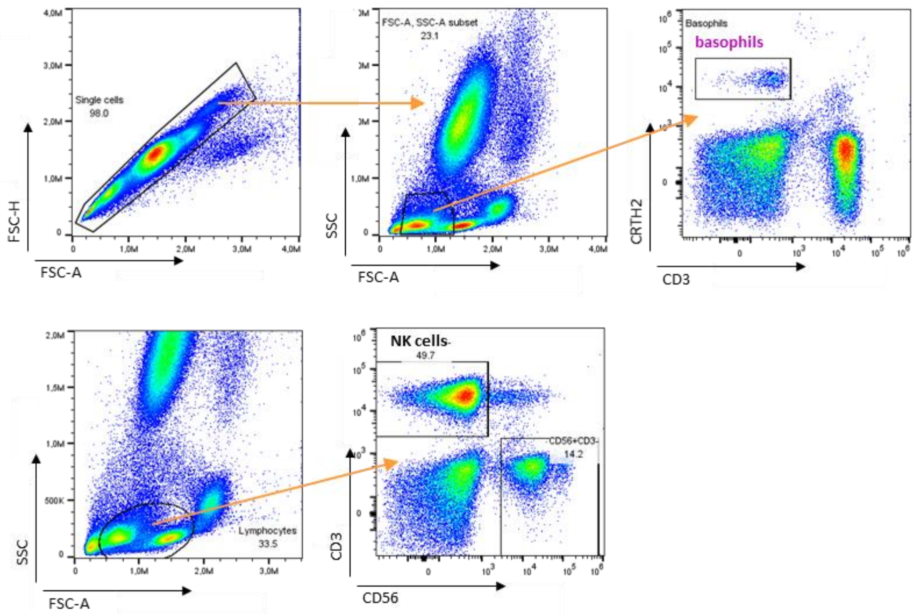

B Gating strategy for NK cells in degranulation assays (see associated Supplementary Figure 1C)

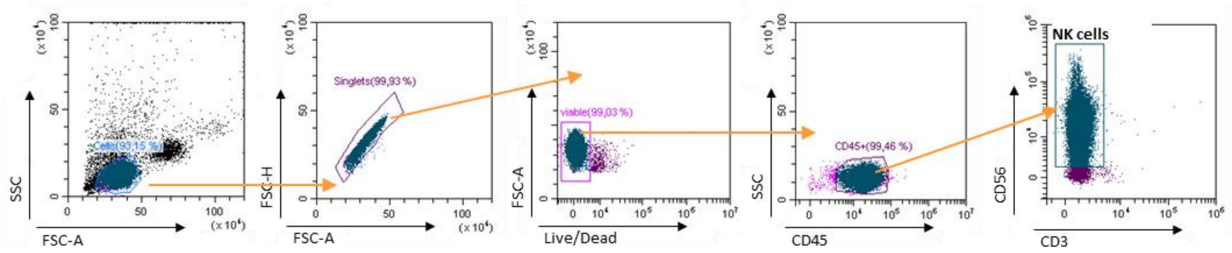

C Gating strategy for EOL-1 cells NK cells after co-culture (see associated Supplementary Figures 1D and 6A)

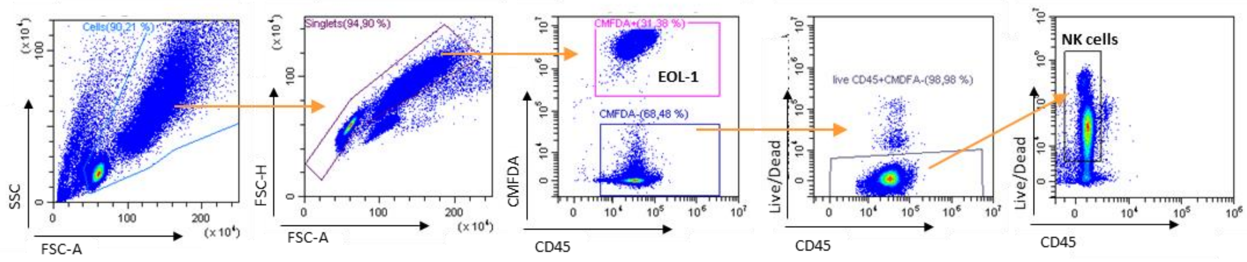

D Gating strategy for NK cells (see associated Supplementary Figure 6B)

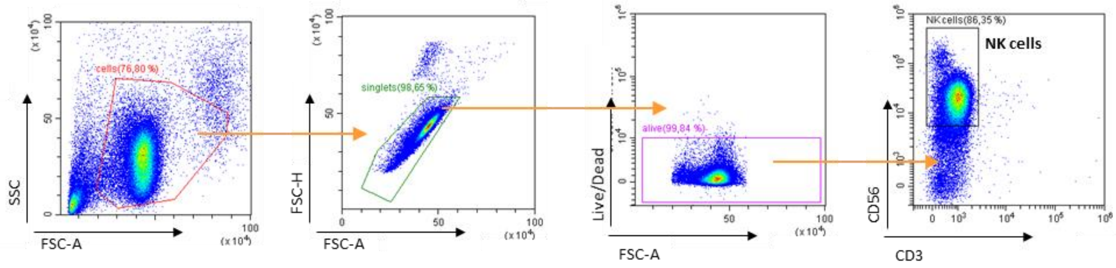

Supplementary Figure 8. Additional gating strategies for NK cells and tumor cells.

A, Gating strategies for basophils and NK cells in whole blood assays, associated with Figure 7C. **B,** Gating strategies for NK cells in degranulation/CD107a assays, associated with Supplementary Figure 1C. **C**, Gating strategies for EOL-1 target cells and NK cells in co-culture assays, associated with Supplementary Figure 1D and Supplementary Figure 6A. **D,** Gating strategies for NK cells, associated with Supplementary Figure 6B.

Supplementary Tables

Supplementary Table 1. Evaluation of AFM28 binding kinetics to human or cynomolgus target and effector antigens.

Receptor	Binding	k_a ($M^{-1}s^{-1}$)	k_d (s^{-1})	K_D (nM)
Human CD16A (158V)	monovalent	1.31 ± 0.07E+6	3.17 ± 0.19E-3	2.60 ± 0.30
	bivalent	1.08 ± 0.14E+6	3.17 ± 0.26E-4	0.30 ± 0.06
Human CD16A (158F)	monovalent	1.78 ± 0.18E+6	3.65 ± 0.21E-3	2.33 ± 0.15
	bivalent	1.02 ± 0.13E+6	2.35 ± 0.37E-4	0.24 ± 0.07
Human CD16B	monovalent	No binding		
	bivalent	n.a.	n.a.	2080 ± 1720
Human CD123	monovalent	9.23 ± 0.86E+5	3.84 ± 0.12E-4	0.42 ± 0.04
	bivalent	7.81 ± 0.40E+5*	5.92 ± 4.90E-6*	0.01 ± 0.01*
Cynomolgus CD16	monovalent	1.53 ± 0.65E+6	5.59 ± 0.37E-3	4.09 ± 1.6
	bivalent**	7.87E+5	3.20E-4	0.41
Cynomolgus CD123	bivalent**	1.73E-6	7.14E-5	0.04

Data are presented as mean and standard deviation (n=3)
k_a, association rate constant; k_d, dissociation rate constant; K_D, dissociation equilibrium constant;
n.a., not applicable
*Kinetic rate constants lie outside of instrument specifications (off-rate too low)
**n=1

Supplementary Table 2. Mutational status of CD123-expressing cell lines used.

Cell line	Origin/disease background	Mutational status (ExPasy Cellosaurus)	Mean CD123 SABC
MOLM-13	AML	FLT3-ITD; MLL-AF9	10610
HNT-34	AML (secondary)	Evi1 overexpressed; Ph+ (t9:22)	9297
KG-1	AML	TP53 mut	9952
EOL-1	acute eosinophilic leukemia	KMT2A partial tandem duplication	7697
TF-1	acute erythroid leukemia	N-Ras; TP53 mut	4932
NOMO-1	AML	TP53 mut; MLL-AF9	5243
THP-1	AML	TP53 mut; MLL-AF9	4982
SKM-1	AML (secondary)	TP53 mut; ASXL-1	2532
OCI-AML3	AML	DNMT3A; NPM1	2380
Kasumi-1	(childhood) AML	RUNX1 (t8:21), TP53 mut, KIT, RAD21	1424
OPM-2	human, multiple myeloma	TP53 mut	negative

AML, acute myeloid leukemia; N/A, not applicable; SABC, specific antibody binding capacity. Data based on n=3-5 experiments.

Supplementary Table 3. Clinical characteristics of BM donor samples from patients with AML.

Patient ID	Sex	Age at BMA	Diagnosis	ELN risk classification 2022	Karyotype [N cells]	Mutations	Blasts in BM (%) cytology	Therapy	Performed assays*
**AML1**	M	76-78	sAML from MDS/MPN	adverse	46,XY,i(17)(q10) [21]	SRSF2, TP53/17p deletion	90	HU	1, 2
**AML2**	M	82-84	sAML from MDS	adverse	46,XY,i(17)(q10) [6], 45,XY,-7,i(17)(q10) [5], 46,XY [9]	SETBP1, SRSF2, TET2	60–80	None	3
**AML3**	M	71-73	sAML from MDS	adverse	46,XY [20]	ASXL1, SF3B1	90	None	1, 2
**AML4**	M	63-65	de novo AML	adverse	46,XY [14], 46,XY,der(2)t(2;13)(?q31;?q31), der(7)t(7;9)(q22;?) [6]	RUNX1	90	None	1, 2
**AML5**	M	75-77	de novo AML	intermediate	46,XY [20]	MLL-PTD	80	None	1, 3, 6
**AML6**	M	71-73	de novo AML	intermediate	46,XY [26]	FLT3D-ITD	Subtotal occupation	None	1, 2, 3
**AML7**	F	79-81	sAML from MPN	adverse	46,XX [26]	IDH2, MPL, RUNX1, SRSF2	40	None	1, 3
**AML8**	M	76-78	de novo AML	intermediate	46,XY [25]	DNMT3A, FLT3-ITD, KMT2A	Subtotal occupation	HU, ARA-C	1, 2, 3, 7
**AML9**	M	69-71	sAML	favorable	46,XY [22]	KRAS, NPMI, RAD21	20	none	4
**AML10**	F	40-42	de novo AML	intermediate	46,XX [20]	BCORL1, IDH2	50	none	4
**AML11**	F	63-65	de novo AML	intermediate	46,XX [26]	CEBPA, JAK2, JAK3, WT1, FLT3-ITD	80	none	4

**AML12**	M	81-83	de novo AML + CLL	intermediate	46,XY [20]	DNMT3A, IDH1, KMT2A-PTD, MYD88 L265P	25	none	4
**AML13**	F	38-40	de novo AML	adverse	46,XX,t(8;21)(q22;q22) [22/25]	ASXL1, RAD21, RUNX1, TET2	50	none	4
**AML14**	F	41-43	de novo AML	adverse	47,XX,+8 [19/30]	BCOR, IDH2, SF3B1, U2AF1, WT1	70-80	none	4
**AML15**	M	75-77	de novo AML	intermediate	46,XY [20]	DNMT3A, GATA2, CBL	30	VEN + AZA	5
**AML16**	F	64-66	de novo AML	N/A	N/A	KMT2A-PTD	80	none	5
**AML17**	F	66-68	de novo AML	adverse	46,XX [20]	ASXL1, CEBPA, SRSF2, STAG2, TET2	80	HU	5
**AML18**	F	51-53	de novo AML	intermediate	49,XX,+8,+18?,+21?[7/15]	KMT2A::MLLT3, BRAF	subtotal occupation	none	5
**AML19**	F	55-57	sAML from MDS	intermediate	46,XX [20]	DDX41	15-30	none	5
**AML20**	F	16-18	de novo AML	adverse	45,X,-X [28], 46,XX [2]	KMT2A-MLLT10, KMT2A-PTD, CUX1, SRSF2	90	none	1
**AML21**	F	77-76	de novo AML	adverse	N/A	TP53	80	HU	1
**AML22**	M	46-48	de novo AML	intermediate	46,XY,t(15;17)(q22;q21) [14]	PML-RARA, FLT3-TKD, FLT3-ITD	80	none	1
**AML23**	F	32-34	de novo AML	intermediate	46,XX [20]	FLT3-ITD	90	none	7
**AML24**	F	73-75	sAML from CMML	favorable	46,XX,t(4;17;17;)(q33;q11;q23),inv(16)(p13q22),ish t(4;17;17;)(NF1-;NF1-;NF1+)	CBFB-MYH11	80	none	7
**AML25**	F	48-50	de novo AML	favorable	46,XX [20]	FLT3-TKD, NPM1	80	none	7

**AML26**	M	56-58	de novo AML	favorable	46,XY [25]	NPM1	total occupation	none	7
**AML27**	F	46-48	de novo AML	intermediate	46,XX [18]	FLT3-ITD, NPM1	90	none	7

AML, acute myeloid leukemia; ARA-C, cytarabine; BM, bone marrow; BMA, bone marrow aspiration; ELN, European LeukemiaNet; HU, hydroxyurea; MPN, myeloproliferative neoplasms; N/A, not applicable; sAML, secondary AML; VEN + AZA, venetoclax + azacytidine.

*1) allogeneic ADCC assay (blasts), 2) allogeneic ADCC assay (LSPCs), 3) Colony-forming unit (CFU) assay, 4) ex-vivo autologous ADCC assay 5) allogeneic ADCC with AML-NK cells, 6) BMMC donor for 5), 7) PD-L1/HLA-ABC induction ADCC assay.

Supplementary Table 4. Clinical characteristics of BM donor samples from patients with MDS.

Patient ID	Sex	Age at BMA	WHO 2016	IPSS-R	Karyotype [N cells]	Mutations	Blasts in BM (%) cytology	Therapy	Performed assays*
**MDS1**	M	76-78	CMML-1	-	46,XY,t(2,2)(p23;q32) [10], 47,XY,t(2,2)(p23;q32),+8 [2], 46,XY[9]	ASXL1, RUNX1, SRSF2	5–9	HU	1, 2, 3
**MDS2**	F	69-71	MDS-EB-2	High	47,XX,+8 [2], 47,XX,+21 [3], 46,XX [17]	CUX1, IDH2, RUNX1, SETBP1, SRSF2	10–20	None	3
**MDS3**	F	58-60	MDS-EB1	Very high	46,XX,del(5)(q21q34) [2], 47,XX,del(5)(q21q34),+8 [18]	None	20	None	1, 2, 3
**MDS4**	F	81-83	CMML-0	-	N/A	N/A	0	None	1, 3
**MDS5**	M	80-82	MDS-EB-1	Int	46,XY [20]	FLT3-ITD, NRAS, SF3B1, STAG2, TET2	5	ESA	1, 2
**MDS6**	M	80-82	MDS-EB-2	Very high	Complex aberrant	TP53	15	None	1, 2
**MDS7**	M	64-66	MDS-EB-1	Int	46,XY [21]	2xDDX41, BCORL1	10	None	2, 3

BM, bone marrow; BMA, bone marrow aspiration; CMML, chronic myelomonocytic leukemia; ESA, erythropoiesis stimulating agent; HU, hydroxyurea; IPSS-R, revised international prognostic scoring system for MDS; MDS, myelodysplastic neoplasms; N/A, not applicable; WHO 2016, WHO classification of MDS 2016.

*1) allogeneic ADCC assay (blasts), 2) allogeneic ADCC assay (LSPCs), 3) Colony-forming unit (CFU) assay, 4) ex-vivo autologous ADCC assay 5) allogeneic ADCC with AML-NK cells, 6) BMMC donor for 5).

Supplementary Table 5. Clinical characteristics of BM samples from healthy donors.

Patient ID	Sex	Age at BMA	Leukocytes (x10E9/L)	Hb (g/dl)	Platelets (x10E9/L)	Performed assays*
**HY1**	F	56-58	10.12	14.8	324	3
**HY2**	M	59-61	7.42	13.9	256	3
**HY3**	F	81-83	6.59	13.6	339	3
**HY4**	F	72-74	7.73	13.7	329	3
**HY5**	M	66-68	5.13	14.9	192	3

BM, bone marrow; BMA, bone marrow aspiration; Hb, hemoglobin; HY, healthy.

*1) allogeneic ADCC assay (blasts), 2) allogeneic ADCC assay (LSPCs), 3) Colony-forming unit (CFU) assay, 4) ex-vivo autologous ADCC assay 5) allogeneic ADCC with AML-NK cells, 6) BMMC donor for 5).

Supplementary Table 6. Antibodies used in this study.

Antigen	Fluorochrome	Clone	Dilution or concentration	Distributor	Cat. No.	RRID
Allogeneic & ex-vivo autologous ADCC assays (blasts & LSPCs)						
**CD33**	BV605	P67.6	1/100	BioLegend	366612	AB_2566405
**CD34**	APC	561	1/200	BioLegend	343608	AB_2228972
**CD38**	BV650	HB-7	1/100	BioLegend	356620	AB_2566233
**CD45**	PerCp-Cy5.5	HI30	1/100	BioLegend	304028	AB_893338
**CD64**	BV650	10.1	1/100	BD Biosciences	740580	AB_2740281
**CD117**	BV785	104D2	1/100	BioLegend	313238	AB_2629837
**CD123**	PE	9F5	1/50	BD Biosciences	555644	AB_396001
Ex-vivo autologous & leukocytes & PD-L1/HLA-ABC induction ADCC assays (NK cells)						
**CD3**	APC/Cy7	HIT3a	1/100	BioLegend	300318	AB_314054
**CD3**	BV510	UCHT1	1/100	BioLegend	300448	AB_2563468
**CD14**	FITC	M5E2	1/100	BioLegend	301804	AB_314186
**CD16**	APC	3G8	1/100	BioLegend	302012	AB_314212
**CD16**	BV650	3G8	1/100	BioLegend	302042	AB_2563801
**CD19**	BV510	HIB19	1/50	BioLegend	302242	AB_2561668
**CD25**	PE/Dazzle 594	BC96	1/50	BioLegend	302646	AB_2734260
**CD45**	BV785	HI30	1/100	BioLegend	304048	AB_2563129

**CD45**	PerCp-Cy5.5	HI30	1/50	BioLegend	304028	AB_893338
**CD56**	APC	5.1H11	1/100	BioLegend	362504	AB_2563913
**CD56**	PE/Cy7	5.1H11	1/50	BioLegend	362510	AB_2563927
**CD69**	PerCp-Cy5.5	FN50	1/50	BioLegend	310926	AB_2074956
**CD107a**	FITC	H4A3	1/10	BioLegend	328606	AB_1186036
**CD123**	Alexa Fluor 647	9F5	1/50	BD Biosciences	563599	AB_2738305
**CD137**	APC	4B4-1	1/50	BioLegend	309810	AB_830672
**CD137**	BV605	4B4-1	1/50	BioLegend	309822	AB_2565997
**CD279 (PD1)**	PE	PD1.3.1.3	1/50	Miltenyi Biotec	130-117-384	AB_2727929
**CD303**	BV421	201A	1/50	BioLegend	354212	AB_2563871
**FcεR1α**	PerCp-Cy5.5	AER-37	1/50	BioLegend	334622	AB_10900257
**IFN-γ**	PE	B27	1/100	BioLegend	506507	AB_315440
			PD-L1/HLA-ABC induction ADCC assays (blasts)			
**CD33**	BV785	WM53	1/100	BioLegend	303428	AB_2650888
**CD34**	PerCp-Cy5.5	561	1/100	BioLegend	343612	AB_2566788
**CD38**	BV650	HB-7	1/100	BioLegend	356620	AB_2566233
**CD45**	BV605	HI30	1/100	BioLegend	304042	AB_2562106
**CD117**	APC-Cy7	104D2	1/100	BioLegend	313228	AB_2566215
**CD123**	PE	9F5	1/50	BD Biosciences	555644	AB_396001

**CD274 (PD-L1)**	APC	29E.2A3	1/100	BioLegend	329708	AB_940360
			Cell line experiments			
**CD64**	PE-Cy7	10.1	1/50	BioLegend	305022	AB_2561584
**CD32**	FITC	FUN-2	1/50	BioLegend	303204	AB_314336
**CD123**	Alexa Fluor 647	6H6	1/100	BioLegend	306023	AB_2562069
			Quantification of cell surface CD123			
**CD123**	N/A	7G3	10 µg/mL	BD Biosciences	554527	AB_395455
			STAT5/6 intracellular staining			
**STAT5**	PE	A17016B.Rec	1/30	BioLegend	936904	AB_2832913
**STAT6**	APC	A15137E	1/30	BioLegend	686018	AB_2728510
**CD33**	BV605	P67.6	1/50	BioLegend	366612	AB_2566405

Supplementary Table 7. Colony-forming unit (CFU) assay manual counts.

Patient sample	Condition				
	CD34+ alone	0 pM	10 pM	100 pM	1000 pM
AML2	167	153	93	89	103
	154	142	90	83	89
	185	156	110	82	77
AML5	820	698	218	47	54
	740	711	173	39	60
	780	768	166	54	68
AML6	116	73	55	18	20
	105	90	46	17	21
	68	88	47	13	12
	50	54	33	14	25
	103	75	24	22	16
AML7	244	247	208	212	132
	282	265	155	140	162
	219	272	140	141	162
AML8	745	748	542	406	296
	770	770	493	378	363
	490	763	482	336	273
MDS1	88	120	49	51	53
	164	139	47	62	60
	118	117	42	38	47
MDS2	261	274	34	76	29
	280	159	46	64	39
	236	170	40	52	44
	185	175	73	63	34
	251	225			

	241	215			
	179	223			
	224	191			
**MDS3**	71	70	62	32	58
	64	40	50	38	37
	44	79	32	33	23
**MDS4**	175	146	44	37	48
	164	145	40	43	62
	127	117	44	29	53
	125	136	35	20	69
	138	112	34	42	50
**MDS7**	61	56	49	55	23
	59	64	62	39	67
	64	58	33		50
**HY1**	70	60	52	50	67
	47	76	54	41	54
	66	68	52	56	50
**HY2**	231	204	182	158	191
	180	184	173	178	178
	211	198	172	150	139
**HY3**	485	479	364	271	343
	391	495	356	260	298
	442	423	296	266	331
**HY4**	161	151	105	110	85
	138	109	112	96	107
	123	164	123	118	106
	134	146	91	84	121
	145	153	138	106	122
	135	147			

	125	148			
	151	154			
	139	179			
	132	144			
	328	345	178	346	205
**HY5**	325	412	259	387	347
	265	364	200	300	215

References

1. Wingert S, Reusch U, Knackmuss S, et al. Preclinical Evaluation of AFM24, a Novel CD16A-Specific Innate Immune Cell Engager Targeting EGFR-Positive Tumors. MAbs. 2021;13(1):1950264.
2. Zhao JX, Yang L, Gu ZN, et al. Stabilization of the single-chain fragment variable by an interdomain disulfide bond and its effect on antibody affinity. Int J Mol Sci. 2010;12(1):1–11.
