# Supplementary material for: The bispecific innate cell engager AFM28 eliminates CD123+ leukemic stem and progenitor cells in AML and MDS
Source: Nat Commun. 2025 Aug 21;16:7793. doi: 10.1038/s41467-025-63069-y (PMC12371032; doi:10.1038/s41467-025-63069-y)
Supplement: Supplementary file 2 — Reporting Summary [file 41467_2025_63069_MOESM2_ESM.pdf]

nature portfolio

Corresponding author(s): Prof. Dr. Daniel Nowak

Last updated by author(s): Jul 24, 2025

Reporting Summary

Nature Portfolio wishes to improve the reproducibility of the work that we publish. This form provides structure for consistency and transparency in reporting. For further information on Nature Portfolio policies, see our Editorial Policies and the Editorial Policy Checklist.

Statistics

For all statistical analyses, confirm that the following items are present in the figure legend, table legend, main text, or Methods section.

n/a	Confirmed	
☐	☒	The exact sample size (*n*) for each experimental group/condition, given as a discrete number and unit of measurement
☐	☒	A statement on whether measurements were taken from distinct samples or whether the same sample was measured repeatedly
☐	☒	The statistical test(s) used AND whether they are one- or two-sided<br>*Only common tests should be described solely by name; describe more complex techniques in the Methods section.*
☒	☐	A description of all covariates tested
☐	☒	A description of any assumptions or corrections, such as tests of normality and adjustment for multiple comparisons
☐	☒	A full description of the statistical parameters including central tendency (e.g. means) or other basic estimates (e.g. regression coefficient) AND variation (e.g. standard deviation) or associated estimates of uncertainty (e.g. confidence intervals)
☐	☒	For null hypothesis testing, the test statistic (e.g. *F*, *t*, *r*) with confidence intervals, effect sizes, degrees of freedom and *P* value noted<br>*Give P values as exact values whenever suitable.*
☒	☐	For Bayesian analysis, information on the choice of priors and Markov chain Monte Carlo settings
☒	☐	For hierarchical and complex designs, identification of the appropriate level for tests and full reporting of outcomes
☒	☐	Estimates of effect sizes (e.g. Cohen's *d*, Pearson's *r*), indicating how they were calculated

Our web collection on statistics for biologists contains articles on many of the points above.

Software and code

Policy information about availability of computer code

Data collection	Flow cytometric data was acquired using the BD FACSDiva software (version 9.0.1). IndiGO 2.0.5.0 software was used for BLI analysis. Interaction kinetics were determined using Biacore T200 Evaluation Software (v3.1).
Data analysis	Flow cytometric data were analyzed using the FlowJo (version 10.8.1) software. Standard statistical analyses were performed using the GraphPad Prism (version 10.2.1) software.

For manuscripts utilizing custom algorithms or software that are central to the research but not yet described in published literature, software must be made available to editors and reviewers. We strongly encourage code deposition in a community repository (e.g. GitHub). See the Nature Portfolio guidelines for submitting code & software for further information.

Data

Policy information about availability of data

All manuscripts must include a data availability statement. This statement should provide the following information, where applicable:

- Accession codes, unique identifiers, or web links for publicly available datasets
- A description of any restrictions on data availability
- For clinical datasets or third party data, please ensure that the statement adheres to our policy

The data generated in this study are provided in the original article/supplementary information/source data file. Unique reagents are available from the authors Jens Pahl (J.Pahl@affimed.com) and Daniel Nowak (daniel.nowak@medma.uni-heidelberg.de).

Research involving human participants, their data, or biological material

Policy information about studies with human participants or human data. See also policy information about sex, gender (identity/presentation), and sexual orientation and race, ethnicity and racism.

Reporting on sex and gender	Information on the sex of participants are available in Supplementary Table 3+4+5. The findings of this study apply to both sexes. Sex and gender were not considered in the study design. Sex was assigned based on karyotype results. Information on the patients' sex was collected as part of their patient file. The patients gave informed written consent for sharing individual level data. In total, primary patient samples of n=12 men and n=15 women with acute myeloid leukemia (AML), n=4 men and n=3 women myelodysplastic neoplasms (MDS), n=2 healthy men and n=3 women were used for this study. Sex- and gender-based analyses were not performed for this study.
Reporting on race, ethnicity, or other socially relevant groupings	The patients' race and ethnicity were not recorded/reported for this study. There were no socially constructed or socially relevant categorization variables used in the study.
Population characteristics	Characteristics of patients are detailed in Supplementary Table 3+4+5 of the manuscript.
Recruitment	Primary patient samples were selected based on a confirmed diagnosis of an AML or an MDS. The study consists of primary patient samples from n=27 AML and n=7 MDS patients, who were treated at the Department of Hematology and Oncology of the Medical Faculty Mannheim, Heidelberg University, Germany. All samples were collected after obtaining the patients' written informed consent and in accordance with the Institutional Review Board 'Ethikkommission II' of the Medical Faculty Mannheim, Heidelberg University, Germany, and the Declaration of Helsinki. There was no bias in selection of the patient samples.
Ethics oversight	The use of primary human materials for research purposes was approved by the Institutional Review Board 'Ethikkommission II' of the Medical Faculty Mannheim of the Heidelberg University.

Note that full information on the approval of the study protocol must also be provided in the manuscript.

Field-specific reporting

Please select the one below that is the best fit for your research. If you are not sure, read the appropriate sections before making your selection.

☒ Life sciences ☐ Behavioural & social sciences ☐ Ecological, evolutionary & environmental sciences

For a reference copy of the document with all sections, see nature.com/documents/nr-reporting-summary-flat.pdf

Life sciences study design

All studies must disclose on these points even when the disclosure is negative.

Sample size	Cell lines: Sample-size calculation for cell lines was not performed. N=10 cell lines were included to provide cell lines with a diverse spectrum of CD123 expression and ADCC susceptibility.<br><br>Primary patient samples: Sample-size calculation for primary patient samples was not performed. The chosen sample size was based on the availability of cryopreserved patient samples stored.<br><br>Mice: Sample-size calculation for mice was not performed. N=8-10 mice per group were chosen to account for experimental variation and handling.<br><br>Cynomolgus: N=3–5 animals per sex per group were used.
Data exclusions	No data were excluded from this study.
Replication	Cell lines: Experiments with cell lines were reproducible and performed at least three times using commercially-obtained cell lines from ATCC and DSMZ as indicated.<br><br>Primary patient samples:<br>Replication: Since the available primary material of the same patient is very limited, the performance of multiple replicates within one experiment is not possible. In this case, reproducibility of the experiments was verified by testing multiple samples from different patients suffering from the same disease (AML or MDS).<br>Reproducibility: The results for the experiments with the primary patient samples were partially non-reproducible in the sense that the results were heterogeneous. This circumstance is explained by and reflects the biological heterogeneity of the samples.<br>Covariates: Due to the limited availability of primary patient samples in MDS and the associated small sample size, adequate statistical control of all covariances is methodologically impossible. The existing biological heterogeneity reflects clinical reality and, as such, is part of the experimental conditions.<br><br>Primary effector cell preparations:<br>Multiple healthy donors were used for e.g. NK cell preparations, ensuring independent experimentation and biological reproducibility.

	Mice: Data were reproducible within the animals tested per group (10 mice per group). Biological effects were reproduced in second model. Age and gender of animals as well as housing conditions were harmonized across groups as per institutional guidelines and authority's approval as indicated.<br><br>Cynomolgus: Data were reproducible within the animals tested per group. No duplicate experiment was performed as per regulations. Age and gender of animals as well as housing conditions were harmonized across groups as per institutional guidelines and authority's approval as indicated.
Randomization	Cell lines: No randomization<br><br>Primary patient samples: Primary patient samples were selected based on the availability and, therefore, not randomized. All samples have been subjected to all treatments within one experiment.<br><br>Animals: Mice (8-10 mice/group) were randomized by body weight across groups. Cynomolgus animals were assigned to dose groups (3-5/sex/group), where possible, based on existing social groups and stratified body weights.
Blinding	Cell lines: Blinding not required since data measurements were not evaluated manually.<br><br>Primary patient samples: The manual evaluation of the CFU assays was performed blinded. For flow cytometric data analysis, the investigator was not blinded. However, bias is not possible because the evaluation settings were the same for all samples.<br><br>Animals: Due to the nature of the treatments and the necessary quality controls, a blinding of the treatments was not possible. However, data measurements were not evaluated manually or were measured according to survival.

Reporting for specific materials, systems and methods

We require information from authors about some types of materials, experimental systems and methods used in many studies. Here, indicate whether each material, system or method listed is relevant to your study. If you are not sure if a list item applies to your research, read the appropriate section before selecting a response.

Materials & experimental systems

n/a	Involved in the study
☐	☒ Antibodies
☐	☒ Eukaryotic cell lines
☒	☐ Palaeontology and archaeology
☐	☒ Animals and other organisms
☒	☐ Clinical data
☒	☐ Dual use research of concern
☒	☐ Plants

Methods

n/a	Involved in the study
☒	☐ ChIP-seq
☐	☒ Flow cytometry
☒	☐ MRI-based neuroimaging

Antibodies

Antibodies used	Brilliant Violet 605™ anti-human CD33 Antibody (BioLegend), Cat#: 366612, clone: P67.6; dilution: 1/100 or 1/50;<br>APC anti-human CD34 Antibody (BioLegend), Cat#: 343608, clone: 561; dilution: 1/200;<br>Brilliant Violet 650™ anti-human CD38 Antibody, Cat#: 356620, clone: HB-7; dilution: 1/100;<br>PerCP/Cyanine5.5 anti-human CD45 Antibody (BioLegend), Cat#: 304028, clone: HI30; dilution: 1/100;<br>BD OptiBuild™ BV650 Mouse Anti-Human CD64 (BD Biosciences), Cat#: 740580, clone: 10.1; dilution: 1/100;<br>Brilliant Violet 785™ anti-human CD117 (c-kit) Antibody (BioLegend), Cat#: 313238, clone: 104D2; dilution: 1/100;<br>BD Pharmingen™ PE Mouse Anti-Human CD123 (BD Biosciences), Cat#: 555644, clone: 9F5; dilution: 1/50;<br>APC/Cyanine7 anti-human CD3 Antibody (BioLegend), Cat#: 300318, clone: HIT3a; dilution: 1/100;<br>Brilliant Violet 510™ anti-human CD3 Antibody (BioLegend), Cat#: 300448, clone: UCHT1; dilution: 1/100;<br>FITC anti-human CD14 Antibody (BioLegend), Cat#: 301804, clone: M5E2; dilution: 1/100;<br>APC anti-human CD16 Antibody (BioLegend), Cat#: 302012, clone: 3G8; dilution: 1/100;<br>Brilliant Violet 650™ anti-human CD16 Antibody (BioLegend), Cat#: 302042, clone: 3G8; dilution: 1/100;<br>Brilliant Violet 510™ anti-human CD19 Antibody (BioLegend), Cat#: 302242, clone: HIB19; dilution: 1/50;<br>PE/Dazzle™ 594 anti-human CD25 Antibody (BioLegend), Cat#: 302646, clone: BC96; dilution: 1/50;<br>Brilliant Violet 785™ anti-human CD45 Antibody (BioLegend), Cat#: 304048, clone: HI30; dilution: 1/100;<br>APC anti-human CD56 (NCAM) Antibody (BioLegend), Cat#: 362504, clone: 5.1H11; dilution: 1/100;<br>PE/Cyanine7 anti-human CD56 (NCAM) Antibody (BioLegend), Cat#: 362510, clone: 5.1H11; dilution: 1/50;<br>PerCP/Cyanine5.5 anti-human CD69 Antibody (BioLegend), Cat#: 310926, clone: FN50; dilution: 1/50;<br>FITC anti-human CD107a (LAMP-1) Antibody (BioLegend), Cat#: 328606, clone: H4A3; dilution: 1/10;<br>BD Pharmingen™ Alexa Fluor® 647 Mouse Anti-Human CD123 (BD Biosciences), Cat#: 563599, clone: 9F5; dilution: 1/100;<br>APC anti-human CD137 (4-1BB) Antibody (BioLegend), Cat#: 309810, clone: 4B4-1; dilution: 1/50;<br>Brilliant Violet 605™ anti-human CD137 (4-1BB) Antibody (BioLegend), Cat#: 309822, clone: 4B4-1; dilution: 1/50;<br>CD279 (PD1) Antibody, anti-human (Miltenyi Biotec), Cat#: 130-117-384, clone: PD1.3.1.3; dilution: 1/50;

Brilliant Violet 421™ anti-human CD303 (BDCA-2) Antibody (BioLegend), Cat#: 354212, clone: 201A; dilution: 1/50;
PerCP/Cyanine5.5 anti-human FcεRIα Antibody (BioLegend), Cat#: 334622, clone: AER-37; dilution: 1/50;
PE anti-human IFN-γ Antibody (BioLegend), Cat#: 506507, clone: B27; dilution: 1/100;
Brilliant Violet 785™ anti-human CD33 Antibody (BioLegend), Cat#: 303428, clone: WM53; dilution: 1/100;
PerCP/Cyanine5.5 anti-human CD34 Antibody (BioLegend), Cat#: 343612, clone: 561; dilution: 1/100;
Brilliant Violet 650™ anti-human CD38 Antibody (BioLegend), Cat#: 356620, clone: HB-7; dilution: 1/100;
Brilliant Violet 605™ anti-human CD45 Antibody (BioLegend), Cat#: 304042, clone: HI30; dilution: 1/100;
APC/Cyanine7 anti-human CD117 (c-kit) Antibody (BioLegend), Cat#: 313228, clone: 104D2; dilution: 1/100;
APC anti-human CD274 (B7-H1, PD-L1) Antibody (BioLegend), Cat#: 329708, clone: 29E.2A3; dilution: 1/100;
PE/Cyanine7 anti-human CD64 Antibody (BioLegend), Cat#: 305022, clone: 10.1; dilution: 1/50;
FITC anti-human CD32 Antibody (BioLegend), Cat#: 303204, clone: FUN-2; dilution: 1/50;
Alexa Fluor® 647 anti-human CD123 Antibody (BioLegend), Cat#: 306023, clone: 6H6; dilution: 1/100;
BD Pharmingen™ Purified Mouse Anti-Human CD123 (BD Biosciences), Cat#: 554527, clone: 7G3; dilution: 10 μg/ml;
PE anti-STAT5 Phospho (Tyr694) Antibody (BioLegend), Cat#: 936904, clone: A17016B.Rec; dilution: 1/30;
APC anti-STAT6 Phospho (Tyr641) Antibody (BioLegend), Cat#: 686018, clone: A15137E; dilution: 1/30.

Validation

We have provided a link for the relevant data sheets for each antibody. The data sheet includes the manufacturer's validation statements, quality control procedures and relevant citations:

Brilliant Violet 605™ anti-human CD33 Antibody (BioLegend), Cat#: 366612, clone: P67.6:
https://www.biolegend.com/en-us/products/brilliant-violet-605-anti-human-cd33-antibody-12255?pdf=true&displayInline=true&leftRightMargin=15&topBottomMargin=15&filename=Brilliant%20Violet%20605%E2%84%A2%20anti-human%20CD33%20Antibody.pdf&v=20240208073156
APC anti-human CD34 Antibody (BioLegend), Cat#: 343608, clone: 561:
https://www.biolegend.com/en-us/products/apc-anti-human-cd34-antibody-6204?pdf=true&displayInline=true&leftRightMargin=15&topBottomMargin=15&filename=APC%20anti-human%20CD34%20Antibody.pdf&v=20230114043032
Brilliant Violet 650™ anti-human CD38 Antibody, Cat#: 356620, clone: HB-7:
https://www.biolegend.com/en-us/products/brilliant-violet-650-anti-human-cd38-antibody-12177?pdf=true&displayInline=true&leftRightMargin=15&topBottomMargin=15&filename=Brilliant%20Violet%20650%E2%84%A2%20anti-human%20CD38%20Antibody.pdf&v=20240207043300
PerCP/Cyanine5.5 anti-human CD45 Antibody (BioLegend), Cat#: 304028, clone: HI30:
https://www.biolegend.com/en-us/products/percp-cyanine5-5-anti-human-cd45-antibody-4240?pdf=true&displayInline=true&leftRightMargin=15&topBottomMargin=15&filename=PerCP/Cyanine5.5%20anti-human%20CD45%20Antibody.pdf&v=20240208073156
BD OptiBuild™ BV650 Mouse Anti-Human CD64 (BD Biosciences), Cat#: 740580, clone: 10.1:
https://www.bdbiosciences.com/content/dam/bdb/products/global/reagents/flow-cytometry-reagents/research-reagents/single-color-antibodies-ruo/740xxx/7405xx/740580_base/pdf/740580.pdf
Brilliant Violet 785™ anti-human CD117 (c-kit) Antibody (BioLegend), Cat#: 313238, clone: 104D2:
https://www.biolegend.com/en-us/products/brilliant-violet-785-anti-human-cd117-c-kit-antibody-13694?pdf=true&displayInline=true&leftRightMargin=15&topBottomMargin=15&filename=Brilliant%20Violet%20785%E2%84%A2%20anti-human%20CD117%20(c-kit)%20Antibody.pdf&v=20240208073156
BD Pharmingen™ PE Mouse Anti-Human CD123 (BD Biosciences), Cat#: 555644, clone: 9F5:
https://www.bdbiosciences.com/content/dam/bdb/products/global/reagents/flow-cytometry-reagents/research-reagents/single-color-antibodies-ruo/555xxx/5556xx/555644_base/pdf/555644.pdf
APC/Cyanine7 anti-human CD3 Antibody (BioLegend), Cat#: 300318, clone: HIT3a;
https://d1spbj2x7qk4bg.cloudfront.net/de-de/products/apc-cyanine7-anti-human-cd3-antibody-1912?displayInline=true&filename=APCCyanine7%20anti-human%20CD3%20Antibody.pdf&leftRightMargin=15&pdf=true&topBottomMargin=15&v=20250409063204
Brilliant Violet 510™ anti-human CD3 Antibody (BioLegend), Cat#: 300448; clone: UCHT1:
https://www.biolegend.com/en-us/products/brilliant-violet-510-anti-human-cd3-antibody-9792?pdf=true&displayInline=true&leftRightMargin=15&topBottomMargin=15&filename=Brilliant%20Violet%20510%E2%84%A2%20anti-human%20CD3%20Antibody.pdf&v=20240208073156
FITC anti-human CD14 Antibody (BioLegend), Cat#: 301804, clone: M5E2:
https://d1spbj2x7qk4bg.cloudfront.net/de-de/products/fitc-anti-human-cd14-antibody-794?displayInline=true&filename=FITC%20anti-human%20CD14%20Antibody.pdf&leftRightMargin=15&pdf=true&topBottomMargin=15&v=20250407123848
APC anti-human CD16 Antibody (BioLegend), Cat#: 302012, clone: 3G8:
https://d1spbj2x7qk4bg.cloudfront.net/de-at/products/apc-anti-human-cd16-antibody-565?displayInline=true&filename=APC%20anti-human%20CD16%20Antibody.pdf&leftRightMargin=15&pdf=true&topBottomMargin=15&v=20250407123848
Brilliant Violet 650™ anti-human CD16 Antibody (BioLegend), Cat#: 302042, clone: 3G8:
https://d1spbj2x7qk4bg.cloudfront.net/de-de/products/brilliant-violet-650-anti-human-cd16-antibody-7655?displayInline=true&filename=Brilliant%20Violet%20650%E2%84%A2%20anti-human%20CD16%20Antibody.pdf&leftRightMargin=15&pdf=true&topBottomMargin=15&v=20250407123848
Brilliant Violet 510™ anti-human CD19 Antibody (BioLegend), Cat#: 302242, clone: HIB19:
https://d1spbj2x7qk4bg.cloudfront.net/de-de/products/brilliant-violet-510-anti-human-cd19-antibody-8004?displayInline=true&filename=Brilliant%20Violet%20510%E2%84%A2%20anti-human%20CD19%20Antibody.pdf&leftRightMargin=15&pdf=true&topBottomMargin=15&v=20250407123848
PE/Dazzle™ 594 anti-human CD25 Antibody (BioLegend), Cat#: 302646, clone: BC96:
https://d1spbj2x7qk4bg.cloudfront.net/de-de/products/pedazzle-594-anti-human-cd25-antibody-15983?displayInline=true&filename=PEDazzle%E2%84%A2%20594%20anti-human%20CD25%20Antibody.pdf&leftRightMargin=15&pdf=true&topBottomMargin=15&v=20250407123848

Brilliant Violet 785™ anti-human CD45 Antibody (BioLegend), Cat#: 304048, clone: HI30:
https://d1spbj2x7qk4bg.cloudfront.net/de-de/products/brilliant-violet-785-anti-human-cd45-antibody-9325?displayInline=true&filename=Brilliant%20Violet%20785%E2%84%A2%20anti-human%20CD45%20Antibody.pdf&leftRightMargin=15&pdf=true&topBottomMargin=15&v=20250407063244
APC anti-human CD56 (NCAM) Antibody (BioLegend), Cat#: 362504, clone: 5.1H11:
https://d1spbj2x7qk4bg.cloudfront.net/de-de/products/apc-anti-human-cd56-ncam-antibody-9941?displayInline=true&filename=APC%20anti-human%20CD56%20(NCAM)%20Antibody.pdf&leftRightMargin=15&pdf=true&topBottomMargin=15&v=20250407123848
PE/Cyanine7 anti-human CD56 (NCAM) Antibody (BioLegend), Cat#: 362510, clone: 5.1H11:
https://d1spbj2x7qk4bg.cloudfront.net/de-de/products/pe-cyanine7-anti-human-cd56-ncam-antibody-9959?displayInline=true&filename=PECyanine7%20anti-human%20CD56%20(NCAM)%20Antibody.pdf&leftRightMargin=15&pdf=true&topBottomMargin=15&v=20250407123848
PerCP/Cyanine5.5 anti-human CD69 Antibody (BioLegend), Cat#: 310926, clone: FN50:
https://d1spbj2x7qk4bg.cloudfront.net/de-de/products/percp-cyanine5-5-anti-human-cd69-antibody-5606?displayInline=true&filename=PerCPCyanine5.5%20anti-human%20CD69%20Antibody.pdf&leftRightMargin=15&pdf=true&topBottomMargin=15&v=20250409063204
FITC anti-human CD107a (LAMP-1) Antibody (BioLegend), Cat#: 328606, clone: H4A3:
https://d1spbj2x7qk4bg.cloudfront.net/de-de/products/fitc-anti-human-cd107a-lamp-1-antibody-4966?displayInline=true&filename=FITC%20anti-human%20CD107a%20(LAMP-1)%20Antibody.pdf&leftRightMargin=15&pdf=true&topBottomMargin=15&v=20250407063244
BD Pharmingen™ Alexa Fluor® 647 Mouse Anti-Human CD123 (BD Biosciences), Cat#: 563599, clone: 9F5:
https://www.bdbiosciences.com/content/dam/bdb/products/global/reagents/flow-cytometry-reagents/research-reagents/single-color-antibodies-ruo/563xxx/5635xx/563599_base/pdf/563599.pdf
APC anti-human CD137 (4-1BB) Antibody (BioLegend), Cat#: 309810, clone: 4B4-1:
https://d1spbj2x7qk4bg.cloudfront.net/de-de/products/apc-anti-human-cd137-4-1bb-antibody-3910?displayInline=true&filename=APC%20anti-human%20CD137%20(4-1BB)%20Antibody.pdf&leftRightMargin=15&pdf=true&topBottomMargin=15&v=20250407063244
Brilliant Violet 605™ anti-human CD137 (4-1BB) Antibody (BioLegend), Cat#: 309822, clone: 4B4-1:
https://d1spbj2x7qk4bg.cloudfront.net/de-de/products/brilliant-violet-605-anti-human-cd137-4-1bb-antibody-12103?displayInline=true&filename=Brilliant%20Violet%20605%E2%84%A2%20anti-human%20CD137%20(4-1BB)%20Antibody.pdf&leftRightMargin=15&pdf=true&topBottomMargin=15&v=20250407123848
CD279 (PD1) Antibody, anti-human (Miltenyi Biotec), Cat#: 130-117-384, clone: PD1.3.1.3:
https://static.miltenyibiotec.com/asset/150655405641/document_ls9tt2ol850a91l2ckur41qo4s?content-disposition=inline
Brilliant Violet 421™ anti-human CD303 (BDCA-2) Antibody (BioLegend), Cat#: 354212, clone: 201A:
https://d1spbj2x7qk4bg.cloudfront.net/de-de/products/brilliant-violet-421-anti-human-cd303-bdca-2-antibody-8709?displayInline=true&filename=Brilliant%20Violet%20421%E2%84%A2%20anti-human%20CD303%20(BDCA-2)%20Antibody.pdf&leftRightMargin=15&pdf=true&topBottomMargin=15&v=20250407123848
PerCP/Cyanine5.5 anti-human FcεRIα Antibody (BioLegend), Cat#: 334622, clone: AER-37:
https://d1spbj2x7qk4bg.cloudfront.net/de-de/products/percp-cyanine5-5-anti-human-fcepsilonrialpha-antibody-7311?displayInline=true&filename=PerCPCyanine5.5%20anti-human%20Fc%CE%B5RI%CE%B1%20Antibody.pdf&leftRightMargin=15&pdf=true&topBottomMargin=15&v=20250409063204
PE anti-human IFN-γ Antibody (BioLegend), Cat#: 506507, clone: B27:
https://d1spbj2x7qk4bg.cloudfront.net/de-de/products/pe-anti-human-ifn-gamma-antibody-1536?displayInline=true&filename=PE%20anti-human%20IFN-%CE%B3%20Antibody.pdf&leftRightMargin=15&pdf=true&topBottomMargin=15&v=20250407123848
Brilliant Violet 785™ anti-human CD33 Antibody (BioLegend), Cat#: 303428, clone: WM53:
https://d1spbj2x7qk4bg.cloudfront.net/de-de/products/brilliant-violet-785-anti-human-cd33-antibody-14138?displayInline=true&filename=Brilliant%20Violet%20785%E2%84%A2%20anti-human%20CD33%20Antibody.pdf&leftRightMargin=15&pdf=true&topBottomMargin=15&v=20250407123848
PerCP/Cyanine5.5 anti-human CD34 Antibody (BioLegend), Cat#: 343612, clone: 561:
https://d1spbj2x7qk4bg.cloudfront.net/de-de/products/percp-cyanine5-5-anti-human-cd34-antibody-12873?displayInline=true&filename=PerCPCyanine5.5%20anti-human%20CD34%20Antibody.pdf&leftRightMargin=15&pdf=true&topBottomMargin=15&v=20250407123848
Brilliant Violet 650™ anti-human CD38 Antibody (BioLegend), Cat#: 356620, clone: HB-7:
https://d1spbj2x7qk4bg.cloudfront.net/de-de/products/brilliant-violet-650-anti-human-cd38-antibody-12177?displayInline=true&filename=Brilliant%20Violet%20650%E2%84%A2%20anti-human%20CD38%20Antibody.pdf&leftRightMargin=15&pdf=true&topBottomMargin=15&v=20250407123848
Brilliant Violet 605™ anti-human CD45 Antibody (BioLegend), Cat#: 304042, clone: HI30:
https://d1spbj2x7qk4bg.cloudfront.net/de-de/products/brilliant-violet-605-anti-human-cd45-antibody-8521?displayInline=true&filename=Brilliant%20Violet%20605%E2%84%A2%20anti-human%20CD45%20Antibody.pdf&leftRightMargin=15&pdf=true&topBottomMargin=15&v=20250407063244
APC/Cyanine7 anti-human CD117 (c-kit) Antibody (BioLegend), Cat#: 313228, clone: 104D2:
https://d1spbj2x7qk4bg.cloudfront.net/de-de/products/apc-cyanine7-anti-human-cd117-c-kit-antibody-12166?displayInline=true&filename=APCCyanine7%20anti-human%20CD117%20(c-kit)%20Antibody.pdf&leftRightMargin=15&pdf=true&topBottomMargin=15&v=20250407063244
APC anti-human CD274 (B7-H1, PD-L1) Antibody (BioLegend), Cat#: 329708, clone: 29E.2A3:
https://d1spbj2x7qk4bg.cloudfront.net/de-de/products/apc-anti-human-cd274-b7-h1-pd-l1-antibody-4376?displayInline=true&filename=APC%20anti-human%20CD274%20(B7-H1,%20PD-L1)%20Antibody.pdf&leftRightMargin=15&pdf=true&topBottomMargin=15&v=20250407063244
PE/Cyanine7 anti-human CD64 Antibody (BioLegend), Cat#: 305022, clone: 10.1:
https://www.biolegend.com/en-us/products/pe-cyanine7-anti-human-cd64-antibody-8194?pdf=true&displayInline=true&leftRightMargin=15&topBottomMargin=15&filename=PE/Cyanine7%20anti-human%20CD64%20Antibody.pdf&v=20240208073156

FITC anti-human CD32 Antibody (BioLegend), Cat#: 303204, clone: FUN-2:
https://www.biolegend.com/en-us/products/fitc-anti-human-cd32-antibody-663?pdf=true&displayInline=true&leftRightMargin=15&topBottomMargin=15&filename=FITC%20anti-human%20CD32%20Antibody.pdf&v=20240208073156
Alexa Fluor® 647 anti-human CD123 Antibody (BioLegend), Cat#: 306023, clone: 6H6:
https://www.biolegend.com/en-us/products/alexa-fluor-647-anti-human-cd123-antibody-8525?pdf=true&displayInline=true&leftRightMargin=15&topBottomMargin=15&filename=Alexa%20Fluor%C2%AE%20647%20anti-human%20CD123%20Antibody.pdf&v=20240208073156
BD Pharmingen™ Purified Mouse Anti-Human CD123 (BD Biosciences), Cat#: 554527, clone: 7G3:
https://www.bdbiosciences.com/content/dam/bdb/products/global/reagents/flow-cytometry-reagents/research-reagents/single-color-antibodies-ruo/554xxx/5545xx/554527_base/pdf/554527.pdf
PE anti-STAT5 Phospho (Tyr694) Antibody (BioLegend), Cat#: 936904, clone: A17016B.Rec:
https://www.biolegend.com/en-us/products/pe-anti-stat5-phospho-tyr694-antibody-19206?pdf=true&displayInline=true&leftRightMargin=15&topBottomMargin=15&filename=PE%20anti-STAT5%20Phospho%20(Tyr694)%20Antibody.pdf&v=20240208073156
APC anti-STAT6 Phospho (Tyr641) Antibody (BioLegend), Cat#: 686018, clone: A15137E:
https://www.biolegend.com/en-us/products/apc-anti-stat6-phospho-tyr641-antibody-15603?pdf=true&displayInline=true&leftRightMargin=15&topBottomMargin=15&filename=APC%20anti-STAT6%20Phospho%20(Tyr641)%20Antibody.pdf&v=20240314073123
PE anti-CRTH2 (CD294) (Biolegend), Cat#: 350106 , clone: BM16
https://www.biolegend.com/en-us/products/pe-anti-human-cd294-crth2-antibody-7381?pdf=true&displayInline=true&leftRightMargin=15&topBottomMargin=15&filename=PE%20anti-human%20CD294%20(CRTH2)%20Antibody.pdf&v=20240201073046

Eukaryotic cell lines

Policy information about cell lines and Sex and Gender in Research

Cell line source(s)	All the suspension cell lines used in the manuscript were purchased by the German Collection of Microorganisms and Cell Cultures (DSMZ): MOLM-13 (ACC-554, RRID:CVCL_2119), KG-1 (ACC-14, RRID:CVCL_0374), EOL-1 (ACC 386, RRID:CVCL_0258), THP-1 (ACC-16, RRID:CVCL_0006), Kasumi-1 (ACC 220, RRID:CVCL_0589), OPM-2 (ACC-50, RRID:CVCL_1625), NOMO-1 (ACC-542, RRID:CVCL_1609), HNT-34 (ACC 600, RRID:CVCL_2071), TF-1 (ACC-334, RRID:CVCL_0559), SKM-1 (ACC-547, RRID:CVCL_0098) and OCI AML3 (ACC-582, RRID:CVCL_1844).
Authentication	Cell lines were not authenticated.
Mycoplasma contamination	All cell lines were tested negative for mycoplasma contamination.
Commonly misidentified lines (See ICLAC register)	No commonly misidentified cell lines according to the ICLAC register were used.

Animals and other research organisms

Policy information about studies involving animals; ARRIVE guidelines recommended for reporting animal research, and Sex and Gender in Research

Laboratory animals	10- to-12-week-old female CB17.SCID hCD16A mice (CB17.SCID-Fcgr4tm2(FCGR3A)/Bcgen) and hIL-15 NOG mice (NOD.Cg-Prkdcscid Il2rgtm1Sug Tg(CMV-IL2/IL15)1-1Jic/JicTac) were purchased from Biocytogen (cat. no. 111174) and Taconic Biosciences (cat. no. 13683-F), respectively. All animals were housed under the following conditions: room temperature; 24±2 °C; relative humidity 50±10 %; light period artificial, 12 hours dark/12 hours light rhythm.<br><br>Purpose-bred cynomolgus monkeys (Macaca fascicularis) of Mauritian origin were selected in order to provide n=18 healthy animals of each sex. At the start of the predose phase, animals weighed 2.4 to 3.5 kg (males) or 2.4 to 3.0 kg (females). The date of birth of each animal ranged from January to December 2019.
Wild animals	No wild animals were used in this study.
Reporting on sex	Only female mice were used in this study.<br>Equal numbers of male and female cynomolgus animals were used.
Field-collected samples	The study did not involve any field-collected samples.
Ethics oversight	Mouse study: Mouse studies were conducted at Experimental Pharmacology & Oncology Berlin-Buch GmbH. All mouse experiments were performed in accordance with the Guidelines for the Welfare and Use of Animals in Cancer Research and of the German Animal Protection Law, and approved by the local responsible authorities (Landesamt für Gesundheit und Soziales (LAGeSo), Berlin, Germany).<br><br>Cynomolgus study: The study was conducted by Labcorp Early Development Services GmbH in Münster (Germany) and was approved by Landesamt für Natur, Umwelt und Verbraucherschutz Nordrhein-Westfalen (LANUV) ethics committee.

Note that full information on the approval of the study protocol must also be provided in the manuscript.

Plants

Seed stocks	n/a
Novel plant genotypes	n/a
Authentication	n/a

Flow Cytometry

Plots

Confirm that:

- [x] The axis labels state the marker and fluorochrome used (e.g. CD4-FITC).
- [x] The axis scales are clearly visible. Include numbers along axes only for bottom left plot of group (a 'group' is an analysis of identical markers).
- [x] All plots are contour plots with outliers or pseudocolor plots.
- [x] A numerical value for number of cells or percentage (with statistics) is provided.

Methodology

Sample preparation

Preparation of cell lines:
Cell lines were harvested from cell culture and washed in PBS with 2% FCS and 0.1% sodium azide prior to staining procedures.

Preparation of primary patient samples:
Primary bone marrow (BM) and peripheral blood (PB) samples from AML (n=15 BM; n=11 PB) and HR-MDS (n=7 BM) patients were obtained from residual diagnostic BM aspirations and blood draws. Healthy BM (n=5) was isolated from femoral heads received after hip replacement surgery. All samples were collected after obtaining the patients' written informed consent and in accordance with the Institutional Review Board of the Medical Faculty Mannheim, Heidelberg University, Germany, and the Declaration of Helsinki. Details on the BM and PB donor characteristics and for which assays the samples were used are provided in Supplementary Tables 3–5. Mononuclear cells from BM (BMMCs) or from patient PB and buffy coats were isolated using red cell lysis. Methods for enrichment and culture of CD34-positive (CD34+) cells from BMMCs and of NK cells from PB are described in the Supplementary Methods.

Preparation of leukocytes from whole blood loops:
Erythrocytes were lysed using FACS lysing solution (BD Biosciences, cat.no. 349202) and the cells were washed with PBS before flow cytometry analysis.

Instrument

BD FACSCelesta (model number: 66093321), Beckman Coulter CytoFlex and Beckman Coulter CytoFlexS

Software

Flow cytometric data was acquired using the BD FACSDiva software (version 9.0.1) or the Beckman Coulter CytExpert Software (version 2.4.0.28).

Cell population abundance

No flourescence-activated cell sorting was performed for this study.

Gating strategy

Gating strategy for cell lines:
Dead cells were excluded based on positive staining for Fixable Viability Dye eFluor 780 . Doublets were excluded using gating for FSC-A and FSC-H.

Gating strategy of blasts and leukemic stem and progenitor cells (LSPCs):
Dead cells were excluded based on the presence of SYTOX Blue staining. Doublets were excluded using gating for FSC-A and FSC-H. Allogenic NK cells were excluded based on the presence of intracellular CMFDA staining. After identification of the SSClowCD45low population consisting of blasts and LSPCs, samples were further analyzed for CD34+ CD38+ CD123+/− blasts and CD34+ CD38− CD117+ CD123+/− LSPCs. The gating strategy is depicted in Supplementary Figure 1.

Boundaries between „positive“ and „negative“ stained cell populations were defined based on isotype and unstained controls.

Human basophils were defined as CRTH2+ CD3- from whole blood loop cultures, and FcεR1a+ CD3− CD14− CD19− CD56− CD303− in leukocyte samples. Cynomolgus basophils were defined as FcεR1a+ CD3− CD14− CD20− CD159a− HLA−DR−. Human pDC were defined as CD303+ CD3− CD14− CD19− CD56− CD303− in leukocyte samples. Cynomolgus pDC were

defined as CD303+ HLA−DR+ CD3− CD14− CD20− CD159a− FcεR1a−.

☒ Tick this box to confirm that a figure exemplifying the gating strategy is provided in the Supplementary Information.
